# Mathematical proof of the Fisher-Escolà *Q* statistical distribution in quantum consciousness modeling

**DOI:** 10.1016/j.csbj.2025.04.025

**Published:** 2025-04-26

**Authors:** Álex Escolà-Gascón, Julián Benito-León

**Affiliations:** a Department of Quantitative Methods and Statistics, Comillas Pontifical University, established by the Holy See, Vatican City State; b Department of Neurology, 12 de Octubre University Hospital, Madrid, Spain; c Instituto de Investigación Sanitaria Hospital 12 de Octubre (Imas12), Madrid, Spain; d Centro de Investigación Biomédica en Red sobre Enfermedades Neurodegenerativas (CIBERNED), Madrid, Spain; e Department of Medicine, Complutense University, Madrid, Spain

**Keywords:** Quantum consciousness, Quantum Fisher Information, Hypothesis testing, *Q* Fisher-Escolà Distribution, Quantum entanglement

## Abstract

Quantum theories have long sought to explain conscious experience, yet their biggest challenge is not conceptual but methodological. A critical gap remains: the lack of statistical tools capable of empirically testing these theories against objective reality. This study introduces and formalizes the *Q* of Fisher-Escolà distribution, the first statistical model to integrate quantum and classical probabilities, enabling robust inferential analysis in neuroscience and consciousness studies. We examined 150 density matrices of entangled states in a 10-qubit quantum system using *IBM*’s quantum supercomputers. Through *maximum likelihood estimation*, we mathematically confirmed that *Q*_Fisher-Escolà_ ∼ *beta*(*a*, *b*, loc, scale). As a key contribution, a novel analytical solution to the *Quantum Fisher Information* (QFI) integral was derived, improving decoherence stability. Additionally, 10⁵ *Monte Carlo* simulations allowed us to establish critical thresholds for α = 0.05, 0.01, 0.001, and 0.0001, while assessing Type I and II error rates. Type I errors appeared in 2–5 % of right-tailed tests at α = 0.05 but approached zero as α decreased. Type II errors occurred in left-tailed tests (1–4 % at α = 0.05) but also diminished with stricter significance levels. In two-tailed tests, both error types remained below 3 %, highlighting the distribution’s robustness. The *Q* of Fisher-Escolà distribution pioneers a statistical framework for modeling quantum-classical interactions in consciousness research. It enables hypothesis testing and predicting subjective experiences, with applications in neuroscience and computational automation. Supported by mathematical proofs and empirical validation, this model advances the integration of quantum probability into neuroscience.

## Highlights

• We introduce *Q* of Fisher-Escolà, a novel statistical distribution designed to test whether conscious experience varies when quantum entanglement effects are considered.
• By solving the integral of the *Quantum Fisher Information Function*, we obtain stable measurements of system susceptibility to quantum decoherence.
• Mitigating quantum decoherence ensures that the transition of information beyond the quantum domain remains unaffected, preserving coherence.
• We present the *Fisher-Escolà paradox*, a decoherence-related variation that may inspire future mathematical applications of quantum mechanics to consciousness.
• The empirical applicability of *Q* of Fisher-Escolà extends to entangled qubit states, enabling structured exposure and systematic stimulus contingencies in implicit learning.
• We provide theoretical tables detailing integrals for *P*(*Q*_Theoretical_≥*Q*_Observed_) at various significance levels, facilitating future research.
• Our work includes statistical decision rules for hypothesis testing and biostatistical guidelines for extending quantum principles beyond their conventional domain.

## Graphical Abstract

**Figure:**
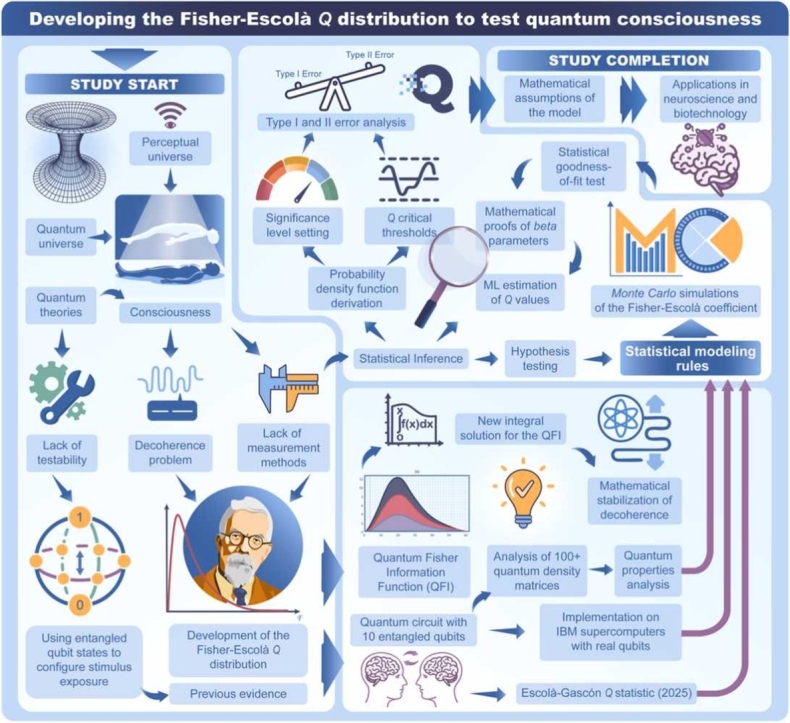

## 1 Introduction

The extent to which conscious experience—or aspects of it—might exhibit quantum-like functioning is a topic of ongoing scientific debate that challenges the limits of perception [37], [38], [39]. Some research lines [11], [33] argue that mental processes cannot be fully explained by classical neuroscience or macroscopic physics because: (a) the mechanistic view of the brain fails to account for the subjectivity and phenomenology of experience [31]; (b) certain biological systems exhibit quantum effects that regulate their survival and homeostasis, such as photosynthesis [75] or bird migration routes [34]; and (c) brain information processing is not restricted to localized sequences in spacetime through chemical reactions or neural networks but also demonstrates nonlocal effects [15], [23] compatible with quantum mechanisms observed in computational processors [6].

For decades, theoretical models and hypotheses have attempted to link quantum properties in certain physiological interactions to the acquisition, processing, and integration of sensory information [21], [22]. Other approaches have applied quantum mathematics to prototype theories [1], leading to the conjecture of quantum cognition [58] and the possibility that thought processes exhibit entangled states [19]. Similarly, von Lucadou [67] and other researchers [69] suggest that certain perceptual experiences beyond the senses may be grounded in the principles of uncertainty and nonlocality [70]. This perspective challenges conventional scientific paradigms, as seen in anomalous cognition and near-death experiences [14], [54], [63], which appear to defy the constraints of orthodox scientific logic. In brief, contemporary research extending quantum phenomena beyond their traditional domain does not lack theoretical rigor, well-founded hypotheses, or conceptual soundness. Instead, the main challenge lies in current methodological limitations. The absence of sufficiently flexible analytical techniques—particularly in statistical modeling—prevents the precise measurement of quantum fluctuations or variations on a broader scale, including behavior, perception, and consciousness.

While traditional science continues to rely on statistical inference and hypothesis testing, researchers who challenge conventional scientific knowledge and epistemology seek to construct a post-materialist science—one that transcends technical limitations that may have hindered progress in studying the *qualia* of conscious experience [35], [8]. Science is more than just a method or a set of technological procedures; it is also a discourse that requires both cosmology and cosmogony to shape its ontological foundations [59]. However, just as it would be unrealistic to attempt crossing the Atlantic Ocean on foot, expecting a paradigm shift in scientific research without providing the necessary resources and means to achieve it is equally unrealistic.

Despite major theoretical advancements in quantum neuroscience, empirical validation remains a critical challenge. This study presents a direct experimental approach, executing 150 quantum circuits on IBM’s supercomputers to extract density matrices from a 10-qubit entangled system, enabling an evaluation of the Fisher-Escolà *Q* distribution. These empirical data provide the first real-world statistical validation of a quantum-classical probabilistic model in neuroscience, bridging the gap between theoretical speculation and empirical analysis.

Building on this foundation, we introduce the Fisher-Escolà *Q* distribution, a novel statistical framework that integrates quantum and classical probabilities, facilitating hypothesis testing in consciousness research. Unlike previous purely theoretical models, this approach enables rigorous statistical inference in quantum neuroscience, providing a practical tool for evaluating quantum-theoretical models of cognition. While primarily mathematical and statistical, its implications extend beyond formal analysis, offering new interdisciplinary perspectives that connect empirical science with conceptual metaphysics.

Furthermore, we detail the experimental origins of the Fisher-Escolà *Q* distribution, outlining its design principles and probabilistic advancements that enhance quantum decoherence stability. We define the *Q* statistic, a system of linear equations designed to quantify hybrid explained variance, integrating quantum entanglement effects with classical data structures. Through *Monte Carlo* simulations, we examine the statistical behavior of key parameters, refining a theoretical Fisher-Escolà *Q* distribution capable of making inferences and generalizing from small samples to larger populations. Finally, we formalize the study’s hypotheses and explore potential applications, aligning our findings with the theoretical models introduced earlier.

### 1.1 Foundations of the original experimental design

The design of the *Q* statistic originated from implicit learning sequences developed under nonlocal physical conditions [15], [12]. The core component of *Q* was the proportion of explained variance extracted from the response patterns of observational units. This explained variance was obtained through *tetracoric* principal axis factorization procedures, represented non-quantum effects, and was denoted as *V*_*k*_. The formulation of the *Q* coefficient aimed to determine which parameters should be included in the system of linear equations to model the potential transfer of quantum entanglement effects to non-quantum patterns. Consequently, *V*_*k*_ had to be combined with the second fundamental component of *Q*, which incorporated quantum mathematics.

At an operational and applied level, the *bipartite* version of *Q* was designed to transfer quantum effects from an entangled two-qubit system (circuit E1) used to establish contingencies between stimuli across 144 experimental trials. Specifically, the binary collapses (0 and 1) of this circuit determined how stimulus associations should occur in an automatically induced classical conditioning procedure. Entanglement occurred within the states of the two qubits, which were in superposition, included a CNOT logic gate, and ended with a *Pauli* operator introducing random axis rotations. If this random rotation was not applied after CNOT activation, the binary collapses of each qubit became identical and constant, allowing for mixed conditions without perfect entanglement. Each of these characteristics was verified through quantum simulators and real physical systems using certified *IBM* quantum supercomputers. The mathematical equations and demonstrations for circuit E1 are detailed in the original report by Escolà-Gascón [12].

**Fig. 1:**
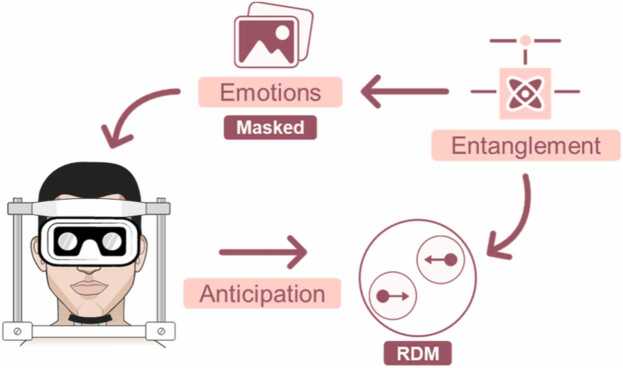
**Schematic and logical diagram of Escolà-Gascón's**[12] **experiment.** This experiment investigated the effects of qubit entanglement on the contingency configuration between emotional stimuli and point movement. The movement of the points (RDM stimuli) became uniform (either leftward or rightward) only after each participant’s anticipation. The central question was whether the covert presentation of emotional stimuli could enable participants to perceive or predict, through quantum-like mechanisms, classically unpredictable stimuli associated with point movement.

The binary collapses mathematically conditioned by preconfigured quantum entanglement were implemented in an experimental design to determine the stimulus pairs forming the classical conditioning contingencies. Participants—monozygotic twin pairs—were placed in sensory isolation conditions and instructed to anticipate stimuli related to moving dots. Since these were contingently related to hidden emotional stimuli via entanglement, participants had to make a conscious decision about the direction in which the moving dots would travel. Their collective decisions formed the response matrix, whose patterns and explained variance are represented by *V*_*k*_. Fig. 1 illustrates the logical process of the experimental design.

The relationships shown in Fig. 1 were designed to test whether configuring stimuli with collapses or quantum measurements conditioned by entanglement could influence the learning process and performance levels of the participants. Simply put, if quantum entanglement had no effect on conscious experience, it should not systematically impact the twins’ learning process. However, findings from Escolà-Gascón [12] indicated that entanglement enhanced both learning and performance. The improvement attributed to entanglement accounted for up to 13 % of the variance in the twins' responses.

### 1.2 The quantum transition: from computers to individuals

One of the most critical and challenging aspects of Escolà-Gascón’s [12] design is understanding how a quantum effect was transferred from circuit E1 (which contained two quantum-entangled qubits) to the decisions made by the twins in the original experiment. This is the central point of the study, as this quantum transition was what the *Q* statistic was intended to model—and what we now aim to generalize through the new probability distribution we are developing.

Although quantum effects are often incorrectly associated with mystical or extraordinary phenomena, the transition we measured was not magical. Circuit E1 generated two binary collapses, each dependent on one of the qubits in the circuit. The entanglement between the qubits occurred prior to these collapses, and it was configured in such a way that it produced latent patterns in the joint sequences of binary collapses. In each run of the circuit, the number of collapses could be programmed as desired. When entanglement was not involved, the collapse sequences were completely random due to the superposition effect induced by the *Hadamard* gate. The control circuit, named C1, included only superposition effects. Circuit E1 also maintained superposition, but immediately afterward incorporated a CNOT gate to induce nonlocal correlations between the two qubits. These nonlocal correlations resulted in entanglement, which was verified by analyzing the structure of the density matrix, obtained through the back-end of *IBM’s Brisbane* quantum computer. The *theory of nonlocal plasticity* (NPT) that supported Escolà-Gascón’s [12] experiment allowed for the conjecture that the collapses derived from previously entangled qubits contained latent patterns bearing a trace of the initial entanglement.

If this framework is correctly understood—and taking into account what was discussed in subsection 1.1.—then in the case that the binary collapses derived from entanglement (and containing latent patterns) were used to configure the presentation of stimulus contingencies later used by individuals to make decisions, it would make sense, or at least be expected, that those same latent patterns—bearing the imprint of entanglement—might also influence the twins’ learning process. What we are trying to clarify here is that if conscious experience involves any form of quantum imprinting (which does *not* mean that consciousness itself is quantum, as suggested by [58], then participants’ learning and its modification due to latent patterns should be proportional to, or show some relationship with, the type of entanglement applied. If this were true, then it would be possible to propose a new equation (the *Q* statistic) capable of mathematically modeling the effects of latent patterns. In a prior exploratory experiment published in *Brain Research Bulletin*, Escolà-Gascón [15] referred to these effects hypothetically as *quantum-like learning*. In both the 2024 and 2025 studies previously published in this journal, latent patterns were indirectly measured using the β parameter of the *Q* statistic, estimated through a Fisherian-derived estimator (as fully explained in Escolà-Gascón’s [12] report). The main limitation of this parameter was that it served as an indirect and *ad hoc* measure of the *Q* statistic. This limitation made it impossible to conduct reliable statistical inferences about whether any quantum effect was actually being transferred via the latent patterns. In this study, we propose to address precisely that limitation by presenting a new probability distribution (comparable to the *Z*, *t*, or the original Fisher’s *F* distribution) that enables the kind of scientific and statistical generalization essential to any experimental research seeking external validity.

In collaboration with Professor Jerome Busemeyer, we are developing a new report in which we mathematically demonstrate the latent patterns present in Escolà-Gascón’s [12] experiment. The same authors of that report confidently affirm that these latent patterns are real and will be published. For readers less familiar with statistical mathematics, we emphasize a key point that underpins the value of all this research: latent quantum-transfer patterns serve to optimize classical learning processes. To state this more boldly, based on the empirical evidence gathered: If a participant without prior training achieved between 69 and 71 correct responses out of 144, the presence of latent patterns—in similarly untrained participants—increased performance to between 82 and 84 correct responses, which is far beyond what would be expected by chance. And this improvement occurred without any prior training, leading us to speculate that introducing a training effect might double or even triple this optimization. Although modest, this optimization is statistically significant and stable according to previous biological findings [12], [15], [42], [58]. Of course, the detection of latent pattern effects in Escolà-Gascón [12] was conducted via explained variance in participants’ responses. Explained variance proportions are standard metrics in classical linear statistics. However, the problem we encountered was the lack of mathematical and statistical tools to model, in a robust, unbiased, and optimal way, the proportions of explained variance attributable to quantum latent patterns. In Escolà-Gascón’s [12] experiment, the *Q* statistic was designed as an *ad hoc* tool usable only for that specific study. However, our aim is to generalize the use of this statistic, and that is what we demonstrate in this report—thereby enabling large-scale reproducibility of this technique in future experiments, which may even yield substantially improved results compared to those in Escolà-Gascón [15], [12]. That is what we now achieve, and what follows is the first probabilistic tool for making such inferences. In the next subsection, we examine the limitations of the original *Q* statistic and explain the steps we have taken in this line of work.

Are we now facing the first stable and mathematically detectable evidence that conscious experience may have a quantum effect? Much has been said on this matter [22], but experimental evidence remains scarce. Overall, our ambition is to provide the statistical tools and methods necessary for advancing quantum biology and consciousness studies—not based on unfounded opinions (even if those opinions are shared by one or many scientists) but grounded in the demonstrable evidence of mathematics and empirical experimentation.

### 1.3 Development of the *Q* statistic and its limitations

Although the experimental design yielded modest results, they were sufficiently significant to develop an initial coefficient—the *Quantum Integrated Multilinear Coefficient* (*Q*)—which integrates the explained variance of monozygotic twins’ responses with that of entanglement. Since 13 % of the variance represented an anomalous or scientifically unexplained weighting, designated in the *Q* algorithm as β, and only provided a preliminary indication of a potential entanglement transition effect, it was necessary for the *Q* coefficient to include explicit indicators to mathematically demonstrate and quantify the extent to which entanglement contributes interpretable and applicable explained variance in implicit learning processes. At this stage, an analysis of the density matrices of both the experimental (entanglement) and control (non-entanglement) groups was conducted to determine whether *Bell*’s inequality was violated. This analysis was complemented by nonlocal correlations and concurrence measures.

When evaluated under quantum mathematical principles, all results indicated that only the qubits in circuit E1 (with entanglement) exhibited entangled states and nonlocal correlations that could not be attributed to hidden variables. This suggested that if the *Q* coefficient was to incorporate entanglement effects that enhanced classical explained variance (*V*_*k*_), it needed to account not only for experimental explained variance and β, but also for *Bell* operators and concurrence, which had to be integrated into the equation. To ensure that *Q* remained within the metric constraints of 0 and 1, a linear integration of *V*_*k*_, β, quantum *concurrence* [26] (denoted as *C*_*Q*_), and *Bell*’s inequality [5]—measured using *S* following the *Clauser-Horne-Shimony-Holt* (CHSH) criterion [9]—was implemented. To guarantee that *Q* could only either remain unchanged (indicating no entanglement-induced transition effect) or increase (signifying the presence of an entanglement effect), the metric adjustment “1+ ” was incorporated into Eq. (1).

(1) $$Q=V_{k}\left(1+\beta C_{Q}S\right)$$

(2) $$\begin{array}{l} H_{0}:\Delta Q=0\\ H_{1}:\Delta Q>0 \end{array}$$

To ensure the occurrence of an entanglement-to-learning transition effect, the condition Δ*Q* > 0 had to be met. This reasoning led Escolà-Gascón [12] to test the null hypothesis of *Q* against 0 (see Eq. (2)) to confirm that deviations were not simply the result of random perturbations or Type I errors. It was assumed that potential Δ*Q* increments-deviations followed the *Student’s t-distribution* model. The results showed that only the experimental group yielded significant findings, providing evidence in favor of a quantum transition effect. It is important to clarify that the equations and formulations concerning concurrence, nonlocal correlations, and *S*, following the CHSH criterion, are presented and demonstrated in Escolà-Gascón [12]. While these findings were promising, three critical issues remained unresolved.

The first of these issues was that Δ*Q* did not necessarily follow the same model as *Q* magnitudes. Although classical increment analyses have traditionally adhered to the *Gaussian model* [13], this assumption did not necessarily hold in the present context. Since *Q* was the first statistic to merge two ontologically distinct sources of variation—quantum and classical—there were valid reasons to question whether its statistical behavior should conform to the predictions of normalized models.

A second, related issue was determining the theoretical model underlying *Q*. If *Q* were merely a classical explained variance, its theoretical distribution could likely be mathematically inferred. However, because *Q* integrates quantum sources of variation and there are no prior references for this type of metrological fusion, its theoretical distribution remained unknown and had to be constructed based on new logical rules that would allow for its mathematical deduction. Developing a new probability model for *Q* would enable hypothesis testing in a way that predicts phenomena integrating both quantum-domain information and general reality, while preserving the coherence conditions required for quantum properties to hold [74], [48].

The third and final issue was that the *Q* statistic was formulated in a way that made it incompatible with multipartite quantum systems (involving three or more qubits). Addressing these three challenges was essential to advancing this line of research and providing the scientific community with a new statistical framework capable of modeling variables exhibiting both quantum and classical behavior.

Quantum statistical advancements are generally focused on sophisticated systems that configure the states of multiple qubits simultaneously [18]. The potential meaning and practical applications of complex quantum systems in human professional activities remain largely unknown [44]. However, their contributions to scientific research have long demonstrated the computational advantages of working with quantum technologies [60].

Following the current trajectory of research on the application of quantum mechanics beyond its original domain [24], defining a statistical rule that enables prediction and inference for decision-making across various fields of knowledge and professional domains would be an extremely useful and, to some extent, necessary methodological resource. From an epistemological perspective, just as it would be meaningless to analyze the behavior of a whale through a microscope, it would be equally inappropriate to apply conventional statistical models to analyze highly precise quantum variations. Aligning statistical techniques with the sensitivity of the information and effects under investigation is the foundational principle that structures the logic of statistical inference.

### 1.4 Current research objectives derived from the *Q* statistic

The objectives of this report were twofold: to develop an optimized version of the *Q* statistic for multipartite quantum systems and construct its theoretical distribution to establish a distribution model capable of representing cognitive and consciousness-related phenomena, integrating both quantum-domain variation and general reality variation. The original version of *Q*, along with its new adaptations, would facilitate the detection of quantum effects in variables or phenomena beyond the scope of quantum mechanics.

Developing specific theoretical distributions for *Q* would mark a significant advancement in incorporating quantum sources of variation into inferential statistical hypothesis testing, ensuring that statistical power is qualitatively sensitive, rather than merely quantitative, to these variations. It would also enable statistical generalizations across populations while accounting for quantum variation effects. Perhaps most notably, it could—for the first time—allow for empirical validation of theoretical frameworks that have thus far remained inaccessible through the scientific method or lacked operational applicability in objective reality.

Regarding this last point, it is important to emphasize that this is not a mere speculative conjecture. The statistical methods developed by engineer and geneticist Sir Ronald A. Fisher [16], [17] transformed numerous disciplines that were initially excluded from the scientific *corpus* because their subjects were considered unsuitable for empirical-positivist scrutiny. Over time, these fields became integral to scientific research, and their contributions to the progression of formal knowledge are now unquestioned. With this historical precedent in mind, we raise a fundamental question: Could the integration of quantum effects into the foundations of theoretical statistics follow a similar trajectory?

## 2 Methods

### 2.1 Analytical process explanation of the study

This section presents the mathematical and statistical criteria used to improve the original *Q* coefficient, following a bidirectional logic: an *outward phase* (simulation-based induction) and a *return phase* (deductive validation). For the outward phase, we conducted 10^5^ *Monte Carlo* simulations with random samples to represent all terms in the new *Q* coefficient equation. Using these simulations, we established statistical rules to derive a simulated distribution, tested various distributional models for *Q*, and estimated a theoretical distribution maximizing *Q* values via the *maximum likelihood criterion*. The return phase began with the derivation of a theoretical distribution with estimated parameters. Through quantile function integration, formally derived and demonstrated, we computed critical thresholds for *Q* at four significance levels and compiled an integral table (Appendix A) detailing probabilities for continuous Q values. These thresholds and tables provided robust criteria for hypothesis testing, presented in the results section.

**Fig. 2:**
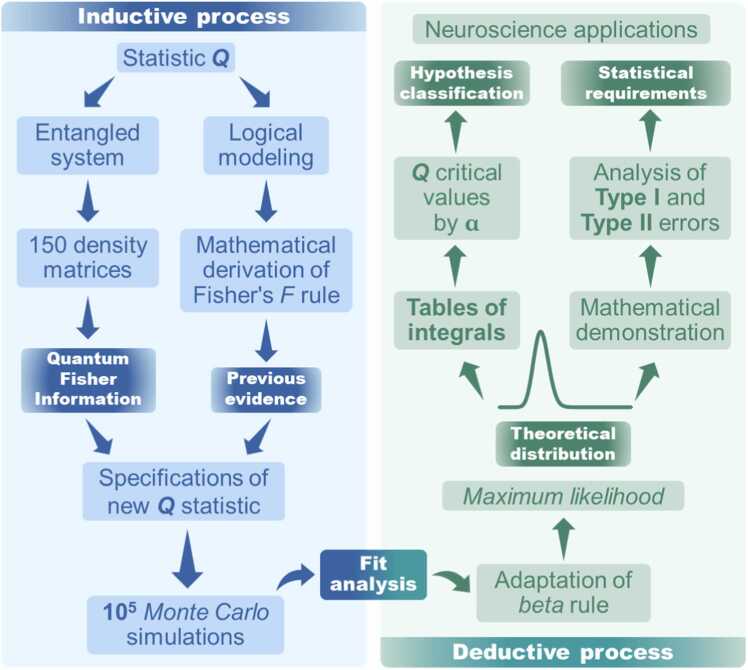
**Summary of the development steps for the*****Q*****of Fisher-Escolà distribution.** This figure provides a conceptual map of the development process, mathematical formulation, and statistical analyses of the Fisher-Escolà *Q* distribution, structured into outward (inductive) and return (deductive) logical paths.

Additionally, we assessed the statistical power of the new distribution, examined Type I and Type II error rates, and included mathematical proofs demonstrating that the new *Q* statistic equation adheres to the *beta* model’s statistical properties. The outward phase was inductive, using simulated random samples, while the return phase was deductive, validating model fit and providing formal proofs of its mathematical soundness. Furthermore, while the outward phase progressed from observational data to theoretical formulation, the return phase transitioned from theoretical development to empirical application, completing the analytical cycle that substantiates our new statistical rule. The diagram in Fig. 2 illustrates and summarizes this methodological process.

The following sections justify the decision criteria and the formulation of *Q*. The results integrate methodological details to maintain clarity and conceptual coherence, avoiding unnecessary separation of execution and analysis.

### 2.2 Multipartite adaptation of the *Q* coefficient

In the original *Q* coefficient (Eq. (1)), the *S* value, derived from the CHSH criterion for analyzing violations of *Bell*’s inequality, is not applicable to systems with three or more qubits. Correcting this initial limitation serves as our starting point for optimizing *Q* and subsequently constructing a new theoretical distribution for implementing hypothesis testing procedures.

Quantum physics offers several alternatives to replace *Bell*’s *S* value under the CHSH criterion, one of the most widely used being the verification of *Mermin* inequality violations [46]. While *Bell*’s inequality violation is established between pairs of quantum states through their density matrices (of size 2² × 2²)—which, in our case, are qubits—*Mermin*’s inequality applies to multi-particle interactions beyond isolated state pairs. The first consequence of this approach is the need to work with density matrices of size 2ⁿ × 2ⁿ, requiring a *Mermin* value based on a multi-state entanglement criterion known as *Greenberger–Horne–Zeilinger* (GHZ) [53]. Notably, GHZ also modifies the metric of *Bell*’s *S* value under the CHSH criterion [9]: whereas *Bell*’s inequality sets an experimental threshold of 2√2 and a theoretical threshold of √16 [56], [57], multipartite systems do not have fixed thresholds or strictly bounded metrics [32]. This introduces mathematical and computational challenges when working under physical entanglement conditions [27].

Although other inequalities derived from *Bell* and *Mermin*’s quantum mathematical rules exist [3], [64], the same problem persists when working with multipartite quantum systems. It is important to clarify that this issue does not stem from errors in the violation of these inequalities—which are, in fact, mathematically solid demonstrations [45]—but rather from two key factors: (a) as a quantum system becomes more multipartite, its complexity increases, making it more difficult to maintain the coherence of its quantum properties [20]; and (b) the more multipartite a quantum system becomes (including more quantum objects or qubits), the more sensitive it is to perturbations when attempting to implement it in physical reality [7]. Both (a) and (b) represent critical challenges that must be addressed if quantum effects are to be extended beyond their native domain.

A possible solution to this problem lies in the logic of the *Quantum Fisher Information coefficient* (hereafter QFI, [17], [55]). This coefficient has multiple formulations and originates from Fisher's equation, which quantifies the amount of variable information contained in a matrix [50]. The QFI of a density matrix (denoted as QFIM) allows us to determine whether a quantum system is susceptible to perturbations that may affect its quantum coherence [2]. The QFIM is designed to indicate the degree of information transition within a quantum system across its different state phases [43]. Thus, the higher the QFIM, the more fluid the system becomes, making it increasingly vulnerable to perturbations. Due to space constraints, we will not present the mathematical formulations of QFIM here. Readers interested in exploring the foundations and applications of QFI and QFIM in greater depth may refer to the works of Wang and Agarwal [71] or Li and Luo [41].

Instead, what is crucial here is to mathematically justify the relationship between QFIM and the *Q* statistic proposed by Escolà-Gascón [12]. In the *Q* equation, the *S* value is no longer functional for multipartite systems and, therefore, must be replaced. However, due to the issues related to decoherence, relying on inequality operators such as *Mermin* does not provide a robust solution either. Our proposal for adapting Eq. (1) consists of removing *S* from the *Q* coefficient and incorporating a QFI function to model system information. Put simply, if we aim to define a parsimonious statistic—one that can efficiently predict quantum effects while integrating general reality effects in highly decoherence-sensitive multipartite conditions—then QFI magnitudes could regulate the effects of decoherence.

However, the *Q* formulation constrains its magnitudes within 0 and 1, as it represents a proportion of explained variance. This restriction prevents QFI from being directly incorporated into Eq. (1). From a logical standpoint, what matters is not a single-point measure of the system's sensitivity to perturbations, but rather the accumulated sensitivity across its executions, allowing for the retention of this sensitivity level.

Mathematically, the solution is to integrate the QFI function, which is what we present next. We define the QFI function as an adaptation of Eq. (1) within our experimental context (for more general formulations, see [40]). Eq. (3) shows the function to be integrated:

(3) $${ℱ}_{Q}\left(\theta \right)=\mathrm{Tr}\left(\rho L^{2}\right)$$

where *L* is the *quantum score operator*, defined by Eq. (4):

(4) $$\frac{\partial \rho \left(\theta \right)}{\partial \theta }=\frac{1}{2}\left(L\rho +\rho L\right)$$

where ρ(θ) has a spectral decomposition, as shown in Eq. (5):

(5) $$\rho \left(\theta \right)=\sum_{i}\lambda_{i}\left(\theta \right)|\psi_{i}\left(\theta \right)\rangle \langle \psi_{i}\left(\theta \right)|$$

Note that λ_*i*_ are eigenvalues and ∣ψ_*i*_〉 are autovectors. From Eq. (5), the QFI takes the form of Eq. (6):

(6) $${ℱ}_{Q}\left(\theta \right)=\sum_{i}\frac{{\left(\partial_{\theta }\lambda_{i}\right)}^{2}}{\lambda_{i}}+2\sum_{i\neq j}\frac{{\left(\lambda_{i}-\lambda_{j}\right)}^{2}}{\lambda_{i}+\lambda_{j}}|\langle \psi_{i}|\partial_{\theta }\psi_{j}\rangle {|}^{2}$$

By specifying the two terms in Eq. (6), we can present Eq. (7) for greater pedagogical clarity:

(7) $${ℱ}_{Q}\left(\theta \right)=\underset{\mathrm{Eigenvalue}\;\mathrm{term}}{\underbrace{\sum_{i}\frac{{\left(\partial_{\theta }\lambda_{i}\right)}^{2}}{\lambda_{i}}}}+\underset{\mathrm{Eigenvector}\;\mathrm{term}}{\underbrace{2\sum_{i\neq j}\frac{{\left(\lambda_{i}-\lambda_{j}\right)}^{2}}{\lambda_{i}+\lambda_{j}}|\langle \psi_{i}|\partial_{\theta }\psi_{j}\rangle {|}^{2}}}$$

As shown, the QFI consists of two additive contributions: one from the eigenvalues and another from the eigenvectors. In scenarios where the parameter dependence is predominantly encoded in the spectrum of the density matrix, the eigenvector term can be neglected, leading to the simplified expression in Eq. (8).

(8) $${ℱ}_{Q}\left(\theta \right)\approx \sum_{i}\frac{{\left(\partial_{\theta }\lambda_{i}\right)}^{2}}{\lambda_{i}}$$

If, instead of considering a discrete set of eigenvalues, we assume that λ(θ) is a differentiable function describing the spectral evolution of the density matrix in parameter space, we can formulate Eq. (9):

(9) $$ds^{2}=\frac{{\left(\partial_{\theta }\lambda \right)}^{2}}{\lambda }d\theta^{2}$$

and comparing Eq. (9) with the *Bures* metric, we verify Eq. (10):

(10) $$ds^{2}={ℱ}_{Q}\left(\theta \right)d\theta^{2}$$

thereafter reaching Eq. (11):

(11) $$f_{Q}\left(\lambda \right)=\frac{{\left(\partial_{\theta }\lambda \right)}^{2}}{\lambda }$$

Therefore, our QFI function, represented in Eq. (11), describes how the spectral structure of ρ(θ) contributes to the quantum information of a multipartite system. From this point, Eq. (11) must be integrated as expressed in Eq. (12):

(12) $$\int_{\lambda_{\min}}^{\lambda_{\max}}\frac{{\left(\partial_{\theta }\lambda \right)}^{2}}{\lambda }d\lambda$$

Our Integral (12) is consistent with the logic of Eq. (8), which allows us to demonstrate Eq. (13):

(13) $${ℱ}_{Q}\left(\theta \right)=\int_{\lambda_{\min}}^{\lambda_{\max}}f_{Q}\left(\lambda \right)d\lambda =\int_{\lambda_{\min}}^{\lambda_{\max}}\frac{{\left(\partial_{\theta }\lambda \right)}^{2}}{\lambda }d\lambda$$

Integral (12) has an analytical solution through *Taylor Series* when *f*(λ) exhibits a smooth behavior. Then, expanding ∂_θ_λ as a *Taylor Series*, we state Eq. (14):

(14) $$\partial_{\theta }\lambda =a_{0}+a_{1}\lambda +a_{2}\lambda^{2}+\ldots$$

and squaring the derivative provides Eq. (15):

(15) $${\left(\partial_{\theta }\lambda \right)}^{2}=a_{0}^{2}+2a_{0}a_{1}\lambda +\left(a_{1}^{2}+2a_{0}a_{2}\right)\lambda^{2}+\ldots$$

Substituting into Integral (12) is expressed in Eq. (16):

(16) $$\int_{\lambda_{\min}}^{\lambda_{\max}}\frac{a_{0}^{2}+2a_{0}a_{1}\lambda +\left(a_{1}^{2}+2a_{0}a_{2}\right)\lambda^{2}+\ldots }{\lambda }d\lambda$$

Breaking it into terms leads to expression (17):

(17) $$a_{0}^{2}\int_{\lambda_{\min}}^{\lambda_{\max}}\frac{d\lambda }{\lambda }+2a_{0}a_{1}\int_{\lambda_{\min}}^{\lambda_{\max}}d\lambda +\left(a_{1}^{2}+2a_{0}a_{2}\right)\int_{\lambda_{\min}}^{\lambda_{\max}}\lambda d\lambda +\ldots$$

By solving each integral, for the *first* term we have Eq. (18):

(18) $$\int_{\lambda_{\min}}^{\lambda_{\max}}\frac{d\lambda }{\lambda }=\;\ln\left(\lambda_{\max}\right)-\;\ln\left(\lambda_{\min}\right)=\;\ln\left(\frac{\lambda_{\max}}{\lambda_{\min}}\right)$$

for the *second* term we have Eq. (19):

(19) $$\int_{\lambda_{\min}}^{\lambda_{\max}}d\lambda =\lambda_{\max}-\lambda_{\min}$$

and for the third term we have Eq. (20):

(20) $$\int_{\lambda_{\min}}^{\lambda_{\max}}\lambda d\lambda =\frac{1}{2}\left(\lambda_{\max^{2}}-\lambda_{\min^{2}}\right)$$

Therefore, final approximation of integral expression is shown in Eq. (21):

(21) $$\int_{\lambda_{\min}}^{\lambda_{\max}}\frac{{\left(\partial_{\theta }\lambda \right)}^{2}}{\lambda }d\lambda \approx a_{0}^{2}\;\ln\left(\frac{\lambda_{\max}}{\lambda_{\min}}\right)+2a_{0}a_{1}\left(\lambda_{\max}-\lambda_{\min}\right)+\left(a_{1}^{2}+2a_{0}a_{2}\right)\frac{1}{2}\left(\lambda_{\max^{2}}-\lambda_{\min^{2}}\right)+\ldots$$

With these demonstrations, we have shown that (12) is an integral with a potentially solvable analytical form, which is essential for our adaptation of the *Q* statistic (see Eq. (1)).

### 2.3 The new Fisher-Escolà *Q* coefficient

So far, in Subsection 2.2, we have justified a mathematical solution to replace *S* in Eq. (1). The proposed integral aims to derive a *cumulative density function* (CDF) or a *probability density function* (PDF) that quantifies the retained sensitivity of a multipartite quantum system to potential perturbations that may compromise the coherence of its quantum properties (e.g., entanglement).

#### 2.3.1 The Fisher-Escolà conceptual paradox

The mathematical paradox in optimizing the original *Q* statistic arises from the fact that classical variances exhibit high sensitivity, making them susceptible to fluctuations. When combined with quantum variances, which are exceedingly small, this can truncate the *Q* calculation, rendering it non-interpretable and non-functional. To explore this issue in more detail: violating *Mermin*’s inequality in a multipartite system requires a highly precise density matrix structure for the qubits, one that cannot be predicted by hidden variables. However, introducing variability to enhance system information increases QFI values, making the system more sensitive to perturbations. If QFI increases excessively, it may disrupt the violation of *Mermin*’s inequality, leading to counterproductive effects. Recall that if *Mermin*’s inequality were not violated, certain hidden variables could predict phase shifts in qubit states, contradicting the intended function of the *Q* statistic. Ideally, the goal is to maximize accumulated information (Integral (12)) while preserving the violation of *Mermin*’s inequality. This challenge explains why quantum mathematical rules have not been fully transferable to inferential statistical procedures beyond strictly quantum domains.

Thus, the paradox can be framed as follows: How can multipartite systems remain highly entangled while maintaining minimal decoherence, ensuring sufficient variability to prevent *Q* from becoming statistically invariant, while still preserving *Mermin*’s inequality violation (which serves as the mathematical verification that qubit states operate in an entangled regime, with nonlocal correlations > 0)?

**Fig. 3:**
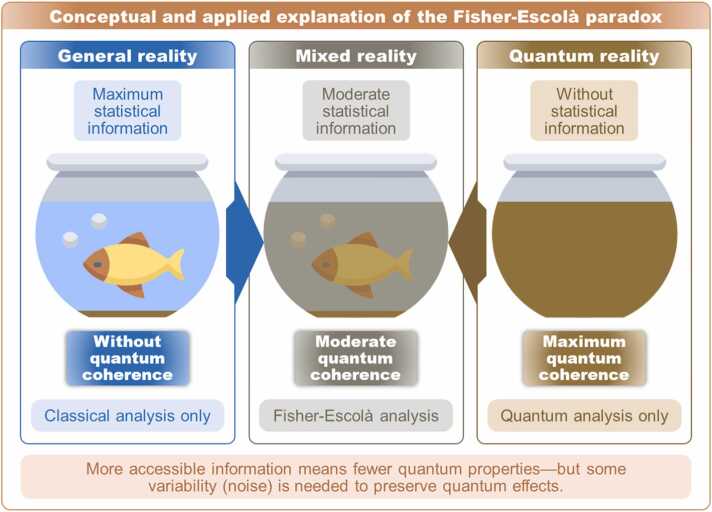
**Applied explanation of the Fisher-Escolà paradox.** This diagram presents a metaphorical interpretation of the Fisher-Escolà paradox. Classical statistical methods fall short when applied to quantum phenomena, as they fail to capture the subtle microvariations inherent at that level. Rather than forcing classical tools onto quantum systems, the proposed approach seeks a middle ground—combining both ontological levels through quantum circuits that incorporate superposition, entanglement, and minimal decoherence (noise). The fish tank metaphor illustrates this contrast: quantum reality appears opaque to classical analysis, while macroscopic reality tends to obscure quantum effects due to excessive deterministic filtering. The aim is to reshape quantum dynamics in a way that makes its information more accessible. One promising strategy involves integrating quantum Fisher information, making it possible to apply this paradox to physical systems beyond the microscopic scale.

To ensure that the Fisher-Escolà paradox is not merely a formal or conceptual construct, we illustrate its application using the example of the three fish tanks shown in Fig. 3. The tank on the left represents a clear, transparent environment where the contents—a fish and two bubbles—are fully visible. In contrast, the tank on the right depicts water that has been stirred to the point of total opacity. If a diver were to submerge into that tank, they would see nothing of what lies inside.

If we understand the fish tank as a non-microscopic physical system, quantum reality—as perceived through classical statistical methods—resembles the murky water: completely opaque. In such a state, maximum coherence might be present precisely because there are no observable distinctions, variations, or differences. However, while the system may remain fully quantum and internally coherent, the information it contains would be entirely inaccessible under the classical framework, which depends on macroscopic differences to detect and interpret data. The core of the paradox is this: in order for quantum effects to become observable outside their native domain, a certain degree of realism must be introduced. In metaphorical terms, the water must become slightly less murky so that the contents—like the fish—can begin to emerge into view. Adding realism to quantum measurements means introducing a level of decoherence, which inevitably reduces or modifies some of the system’s quantum properties.

This paradox was formally noted by Tegmark [65] and later discussed by other researchers [66]. However, no successful attempts have been documented to resolve quantum coherence loss within an operational inferential statistical framework. Addressing this issue in the *Q* statistic enhancement requires solving two key problems: (a) designing a multipartite quantum system that can be executed repeatedly, generating as many density matrices as possible while incorporating maximally entangled states with a controlled degree of noise or variation to prevent *Q* from becoming invariant, all while maintaining *Mermin*’s inequality violation; and (b) resolving the metric constraints of Integral (12), since the maximum and minimum values of λ are unbounded, requiring an adjustment to fit *Q*’s bounded metric. Subsections 2.3.2 and 2.3.3 provide solutions to these challenges.

As illustrated in Fig. 3, the paradox suggests that—counterintuitively—quantum effects require a minimal amount of decoherence (or realism) to be perceived beyond their quantum framework. The proposed solution involves an integral-based formalism that allows for the stabilization of quantum decoherence: introducing just enough realism to make quantum information accessible, without entirely eliminating its quantum characteristics. This balance is represented by the central tank, where the water is slightly clouded but still transparent enough to distinguish its contents—thanks to contextual decoherences that make interpretation possible.

#### 2.3.2 Multipartite and entangled EM1 circuit

**Fig. 4:**
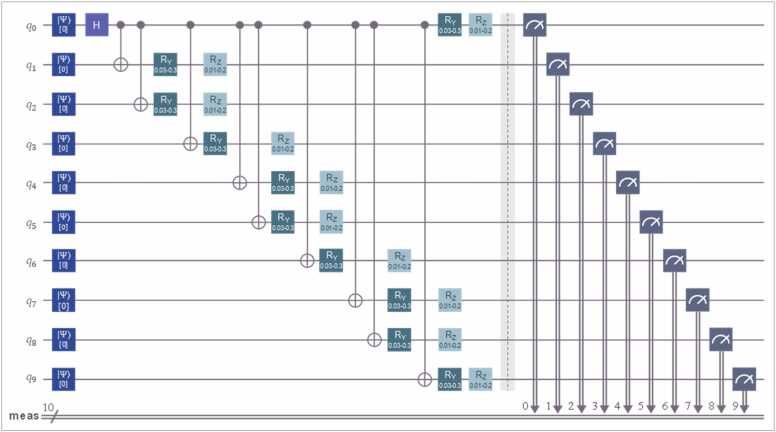
**EM1 Circuit: 10-Qubit System with Successive CNOT Gates.** The EM1 circuit consists of 10 qubits with successive CNOT gates that generate non-local correlations, inducing entangled states. The *R* gates are rotational gates that modulate entanglement levels. Measurements were binary, with one collapse per qubit, ensuring the most precise configuration. However, statistically, longer sequences can be produced, extending beyond the 10-qubit limit. The effectiveness of entanglement effects on collapses depends on the qubit states. By logical deduction, the highest variability in defining the theoretical distribution of *Q*_Fisher-Escolà_ is achieved when each qubit undergoes exactly one collapse.

We designed a multipartite quantum circuit-system with 10 qubits, initializing the system in state 0 and applying a *Hadamard gate* (*H*) to the first qubit to generate quantum superposition. Nine CNOT gates were then applied from the first qubit (*q*_1_) to the ninth (*q*_9_), with *q*_0_ as the control qubit. These CNOT gates induced nonlocal correlations between qubit states, allowing for the generation of a maximally entangled system that systematically violates *Mermin*’s inequality. To prevent the system from becoming excessively coherent or invariant (from a classical perspective), we introduced *Pauli* operators that applied small random rotations to the states. Specifically, *R*_*y*_ fluctuations ranged from 0.03 to 0.3, while *R*_*z*_ fluctuations varied between 0.01 and 0.2. These controlled-noise logic gates were interspersed between the CNOT gates. The circuit concluded with a binary collapse of the qubits (0 or 1). Fig. 4 provides a visual representation of the circuit configuration.

We designated this circuit as EM1 and successfully executed it 150 times on real physical quantum systems, using *IBM*’s *Brisbane* and *Sherbrooke* quantum supercomputers. This is a key detail, as we obtained 150 density matrices, each with dimensions 2¹⁰ × 2¹⁰, extracted directly from the back-ends of these supercomputers. Thus, these density matrices were not numerical simulations, but mathematical reconstructions derived from real empirical data. Each of these 150 executions was performed manually, one at a time, in sequential order, simultaneously recording the matrices corresponding to the collapses as well as the average qubit reaction times from each supercomputer. Had we opted for simulations instead of real executions, the number of iterations would have been significantly higher. However, *IBM*’s quantum supercomputer usage restrictions prevent individual users from extracting thousands of density matrices for qubit states. Nonetheless, this limitation did not prevent us from conducting an initial statistical approximation.

Within behavioral sciences and neuroscience, our approach is so novel that previous scientific literature provides little information on how many density matrices researchers typically use in experimental designs involving quantum qubit systems. It is important to clarify that the number of density matrices is not equivalent to sample size (*N*). A single density matrix can generate multiple sequences of binary collapses attributable to qubits (as demonstrated by [15], [12]). The number of density matrices does not depend on *N* but rather on the experimental conditions assigned to the analyzed groups. If each group in an experimental research design is exposed to different conditions, then, for ontological reasons, distinct qubit circuit systems should be used.

For example, Escolà-Gascón [12] distinguished two groups: the experimental group, randomly assigned, operated with a two-qubit entangled circuit, while the control group used a circuit with independently functioning qubits. Additionally, the number of density matrices may also depend on the number of experiments conducted within a single study. If an experiment involved two independent groups and was replicated in two separate experiments, four distinct types of density matrices would be obtained. Thus, the number of *N* sequences of collapses could be easily adjusted for each density matrix.

Our 150 density matrices were generated from the same EM1 circuit and obtained by programming its executions in *Python 3.9.21*. Each density matrix corresponded to an individual execution of EM1 on *IBM*’s quantum supercomputers. Specifically, 75 executions were performed on *IBM Brisbane* and 75 on *IBM Sherbrooke*. This combination of real quantum systems, while keeping EM1 unchanged, allows for an analysis—if necessary—of whether supercomputer-specific errors affected the quantum properties of the qubit states. This can be evaluated, for example, by analyzing qubit reaction times in each execution. If temporal coherence remains low, no perturbations can be attributed to the hardware characteristics of *IBM*’s supercomputers (each of which supports up to 127 simultaneous qubits).

This qubit limit is unrelated to the question of how many qubits should be included in each circuit designed and applied by researchers. In biological and behavioral sciences, we have found no real applications of circuits exceeding 10 qubits (although some theoretical circuits propose models with more than 10 qubits, their applicability in neuroscience, biology, and behavioral sciences remains uncertain). As previously mentioned, the use of qubits outside computer science to model biological or behavioral phenomena is a highly innovative approach with more uncertainties than certainties. This underscores the need for further scientific reports that provide expanded and refined results, as we aim to do here.

From a mathematical perspective, we now present the framework used to assess the extent to which the EM1 circuit maintained entangled qubit states. To evaluate *Mermin*’s inequality, we applied operator (22):

(22) $$M_{10}=\frac{1}{2}\left[\overset{10}{\underset{j=1}{⊗}}\left(\sigma_{x}^{\left(j\right)}+i\sigma_{y}^{\left(j\right)}\right)+\overset{10}{\underset{j=1}{⊗}}\left(\sigma_{x}^{\left(j\right)}-i\sigma_{y}^{\left(j\right)}\right)\right]$$

where ⊗ denotes the tensor product. Both σ_*x*_ and σ_y_ are the *Pauli* matrices defined in (23), (24).

(23) $$\sigma_{x}=\left(\begin{array}{cc} 0 & 1\\ 1 & 0 \end{array}\right)$$

and

(24) $$\sigma_{y}=\left(\begin{array}{cc} 0 & -i\\ i & 0 \end{array}\right)$$

From this point onward, the *Mermin* expected operator was computed using Eq. (25):

(25) $$\left\langle M_{10}\right\rangle =\mathrm{Tr}\left(M_{10}\rho \right)$$

where ρ= ∣ψ〉〈ψ∣ is the ρ of the prepared state (Eq. (26)):

(26) $$\left|\psi \right\rangle =\overset{10}{\underset{j=1}{⊗}}\left[R_{z}^{\left(j\right)}\left(\Phi_{j}\right)R_{y}^{\left(j\right)}\left(\theta_{j}\right)\right]\left|{\mathrm{GHZ}}_{10}\right\rangle$$

where *R* is rotations of the circuit.

Likewise, θ_*j*_ is the angle range, fixed as Φ_*j*_ ∈ [0, π/20], ensuring small *rotations* (*R*), and the GHZ state is a specific quantum state for multiple quantum objects (qubits in our case) that ensure entanglement. The GHZ can be formulated as shown in Eq. (27):

(27) $$|{\mathrm{GHZ}}_{10}\rangle =\frac{1}{\sqrt{2}}\left({|0\rangle }^{⊗10}+{|1\rangle }^{⊗10}\right)$$

In Eq. (26), the rotations were controlled and are expressed as shown in the mathematical process (28):

(28) $$\begin{array}{l} \mathrm{Hadamard}\Rightarrow |\psi_{1}\rangle =\frac{1}{\sqrt{2}}\left(\left|0\rangle +|1\right\rangle \right)⊗|0\rangle^{⊗9}\\ \mathrm{CNOT}\Rightarrow |\psi_{2}\rangle =\frac{1}{\sqrt{2}}\left(\left|0000000000\rangle +|1111111111\right\rangle \right)\\ R_{y}\left(\theta \right)=\left[\begin{array}{cc} \cos\left(\theta /2\right) & -\;\sin\left(\theta /2\right)\\ \sin\left(\theta /2\right) & \cos\left(\theta /2\right) \end{array}\right]\\ R_{z}\left(\phi \right)=\left[\begin{array}{cc} e^{-i\phi /2} & 0\\ 0 & e^{i\phi /2} \end{array}\right] \end{array}$$

These rotations introduce phase shifts in the qubit states, leading to slight but controlled alterations in their entanglement, as previously noted (see Eq. (29)).

(29) $$\begin{array}{c} |\psi_{3}\rangle =\frac{1}{\sqrt{2}}(R_{y}\left(\theta_{0}\right)R_{z}\left(\phi_{0}\right)|0000000000\rangle +\\ R_{y}\left(\theta_{1}\right)R_{z}\left(\phi_{1}\right)|1111111111\rangle ) \end{array}$$

(22), (23), (24), (25), (26), (29) clearly illustrate the logic of the quantum circuit or system in Fig. 4. Please note that the process in Eq. (28) is presented as an example of how it can be applied to the remaining qubits. *Global Quantum Concurrence* (*C*_*Q*_) is defined in Eq. (30), providing a measure of global concurrence levels.

(30) $$C_{G}\left(\rho \right)=\sqrt{\frac{2^{N}}{2^{N}-1}\left(1-\mathrm{Tr}\left(\rho^{2}\right)\right)}$$

Eq. (30) is widely used in this type of circuit and provides an approximation of the entanglement levels of qubit states for each execution and density matrix [47], [52].

#### 2.3.3 Adjustment of the metric of Integral (12)

At this stage, we present the corrections made to Integral (12) to adjust it to a bounded metric compatible with the probabilistic characteristics (0−1) of the *Q* coefficient. According to Popescu & Rohrlich [57], the *S* value in *Bell*’s CHSH criterion can theoretically range between 0 and 4. Multiplying this value by concurrence (0−1), the β weight from Eq. (1), and *V*_*k*_ allowed us to model the original variance based on entanglement effects and nonlocal correlations, ensuring a proportional increase within the 0–1 range. Since the new version of *Q* must remain within these limits, as it represents explained variance, the most practical approach was to have Integral (12) replace *S*. However, Integral (12) does not follow the same metric scale as *S*. This metric discrepancy led us to standardize Integral (12) by dividing it by the maximum value of *f*(λ). This procedure, presented in Eq. (31), is referred to as *I*_*Q*_:

(31) $$I_{Q}=\frac{\int_{\lambda_{\min}}^{\lambda_{\max}}\frac{{\left(\partial_{\theta }\lambda \right)}^{2}}{\lambda }d\lambda }{\max\;f\left(\lambda \right)}$$

Eq. (31) allowed the values of Integral (12) to be constrained within the 0–1 range. When this *I*_*Q*_ result was multiplied by four, it was weighted according to the metric demonstrated theoretically by Popescu & Rohrlich [57]. Therefore, a first optimized version of Eq. (1) is presented in Eq. (32):

(32) $$Q_{\mathrm{Fisher}-\mathrm{Escol}à}=V_{k}\left(1+\beta C_{Q}\left(4I_{Q}\right)\right)$$

Since Eq. (32) incorporates an integral of the QFI function (adapted from Ronald A. Fisher’s method, see [17]) and is implemented in the *Q* coefficient (formulated by Escolà-Gascón [12]), it was designated as the *Fisher-Escolà Q* coefficient. Eq. (32) preserves the properties of the original *Q* statistic, while its new formulation enables *Q* to be recalculated for quantum systems with up to 10 qubits, ensuring the metric remains within the 0–1 range.

## 3 Results

### 3.1 *Monte Carlo* simulations

One of the key challenges in this report was determining whether the values of *Q* and *Q*_Fisher-Escolà_, which integrate two distinct ontological sources of variability—quantum mechanics and general reality—follow a unique statistical rule rather than conforming to established probability models. This question is neither unfounded nor arbitrary; given the lack of prior evidence on the effects of combining these two ontological sources of variation, a scientific approach requires investigating this possibility.

To establish the theoretical foundation for *Q*_Fisher-Escolà_, we must mathematically formalize and demonstrate how to model the various terms in Eq. (32). Since Eq. (32) can be decomposed into two components—a *classical* component (*V*_*k*_ and β) and a *quantum* component (*C*_*Q*_ and *I*_*Q*_)—we first develop the logical framework for modeling the classical terms of *Q*_Fisher-Escolà_ (Subsections 3.1.1 and 3.1.2), followed by the derivation of the theoretical values for the quantum component (Subsection 3.2).

#### 3.1.1 Monte Carlo simulations for the terms V_k_ and beta

Starting with *V*_*k*_ and β—both representing proportions of explained variance, the former derived from *exploratory factor analysis* (EFA) and the latter from the partial η² coefficient—we have mathematical grounds to deduce the probability distribution each follows.

Focusing first on *V*_*k*_, it represents explained variance obtained from *eigenvalues* (λ) corresponding to *latent factors* (*k*) extracted from the participants' response matrix. This distinction separates systematic variance (associated with factors) from residual variance (linked to uncertainty). By considering λ_residual_ and λ_*k*_, we can define their mathematical relationship in a way that yields a statistic expected to follow an *F*-distribution, *F*(*d*₁, *d*₂), making it a form of Fisher’s statistic (see Eq. (33)):

(33) $$\frac{\sum \lambda_{k}}{\sum \lambda_{\mathrm{residual}}}=F∼F\left(d_{1},d_{2}\right)$$

**Fig. 5:**
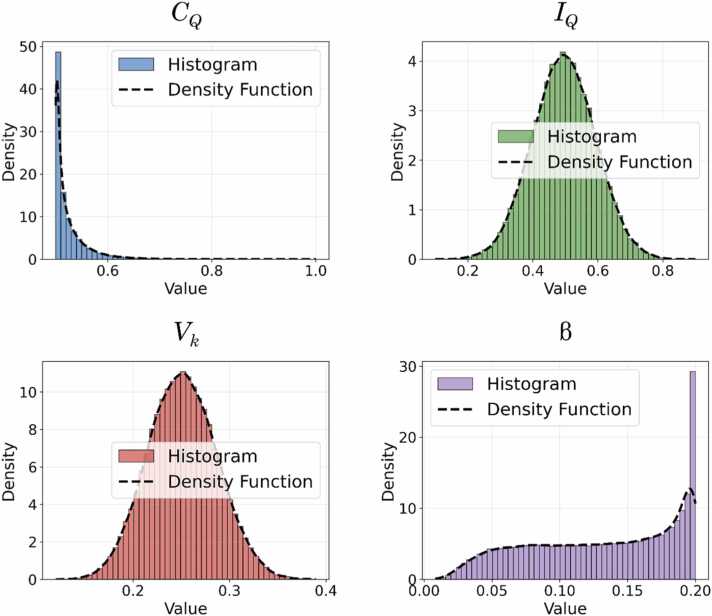
**Descriptive Statistics of*****Monte Carlo*****Simulations.** We conducted 10^5^*Monte Carlo* simulations using the *Kernel Density Estimation* (KDE) method to estimate the density functions of the histograms. Below are the descriptive statistics for each distribution: (a) global concurrency: Mean = 0.524373, Standard Deviation = 0.036046, Skewness = 3.176115, and Kurtosis = 15.686106. (b) *I*_*Q*_: Mean = 0.495488, Standard Deviation = 0.096909, Skewness = -0.004207, and Kurtosis = -0.034250. (c) *V*_*k*_: Mean = 0.251313, Standard Deviation = 0.035653, Skewness = 0.070194, and Kurtosis = -0.128742. (d) β: Mean = 0.130817, Standard Deviation = 0.053233, Skewness = -0.315361, and Kurtosis = -1.176788. These values provide insight into the distributional properties of the simulated data, highlighting variations in dispersion, asymmetry, and tail behavior.

If the relationship between λ_residual_ and λ_*k*_ holds, then *V*_*k*_ follows an *F*-distribution, *V*_*k*_ ∼ *F*(*N* − *K*, *K*−1), where *d*₁ = *N* − *K* and *d*₂ = *K*−1. For β, being derived from partial η², we establish the following equivalence (Eq. (34)) between this coefficient and the *F*-statistic:

(34) $$\begin{array}{c} \beta =\eta_{p}^{2}=\frac{SS_{\mathrm{factor}}}{SS_{\mathrm{factor}}+SS_{\mathrm{error}}}\propto F=\frac{MS_{\mathrm{factor}}}{MS_{\mathrm{error}}}=\\ \frac{SS_{\mathrm{factor}}/\left(J-1\right)}{SS_{\mathrm{error}}/\left(N-J\right)}∼F\left(d_{1},d_{2}\right) \end{array}$$

where *SS* represents sum of squares and *MS* denotes mean square. If Eq. (34) holds, then β ∼ *F*(*N* − *J*, *J*−1), where *J* is the number of groups defined by the experimental conditions. Here, *d*₁ = *N* − *J* and *d*₂ = *J*−1. Considering the experimental values reported by Escolà-Gascón [12], we have *N* = 106, *J* = 2, and *K* = 38. Thus, we obtain *V*_*k*_ ∼ *F*(68, 37) and β ∼ *F*(104, 1). Using these values, we estimate 10⁵ *Monte Carlo* simulations with completely random samples. Fig. 5 illustrates the distributions for all terms in Eq. (32).

#### 3.1.2 Monte Carlo simulations for the terms C_Q_ and I_Q_

At this stage, we leveraged the 150 empirically executed density matrices from *IBM*’s quantum supercomputers (see Subsection 2.3.2) under near-perfect entanglement conditions. The calculations of *C*_*Q*_ (30) and *I*_*Q*_ (31) based on these 150 density matrices yielded ideal values for a highly entangled system (*C*_*Q*_ ≈ 0.9999; *I*_*Q*_ ≈ 0.5001). The fact that *I*_*Q*_ was close to 0.5 indicates that the system contained processable and extractable quantum information.

One might assume that an *I*_*Q*_ ∼ 1 would be ideal, but this interpretation is incorrect. An excessively high *I*_*Q*_ would indicate that the system is highly sensitive to random perturbations, potentially disrupting quantum entanglement properties. Conversely, an *I*_*Q*_ ∼ 0 would also be undesirable, as it would suggest that the density matrix contains hermetic or non-extractable information, making it inaccessible outside quantum physics. Therefore, an *I*_*Q*_ value around 0.5 represents an optimal balance. This interpretative criterion suggests that *I*_*Q*_ follows Fisher’s transformation, which is adjustable to a normal distribution *N*(μ, σ).

*Concurrence* (*C*_*Q*_) measures the degree of entanglement in a system, where *C*_*Q*_ > 0.5 indicates a partially entangled system [73]. The ideal value for near-perfect entanglement is *C*_*Q*_ ∼ 1, consistent with the values obtained from our circuit. For concurrence, we applied an *F*(149, 9) model, where concurrence values closest to 0.5 were the most probable, accounting for possible system decoherence induced by perturbations.

We conducted 10⁵ *Monte Carlo* simulations using random samples based on the specifications and characteristics outlined earlier. These 10⁵ values for each term were adjusted to align with the 150 density matrices obtained from qubit states in the EM1 circuit. The descriptive statistical properties of these distributions are presented in the legend of Fig. 5. They are not included in the main text, as our primary focus was the construction of a theoretical statistical model for the Fisher-Escolà *Q* coefficient.

Additionally, the raw data matrix containing all *Monte Carlo* simulations for each term in Eq. (32) is available as supplementary *Excel* files accompanying this report (see "Monte_Carlo_Vk_beta" and "Monte_Carlo_Cq_Iq").

#### 3.1.3 Monte Carlo simulations for the Q_Fisher-Escolà_

Having obtained *Monte Carlo* estimates for all terms of the Fisher-Escolà coefficient, the next step was to generate theoretical *Q* estimates to derive specific probabilities through integration procedures. The goal of constructing a theoretical model was to enable statistically straightforward hypothesis testing to determine the proportion of explained variance in *Q*_Fisher-Escolà_—which integrates both general and quantum sources of variability—that aligns with a new random model. To obtain theoretical *Q* values, we followed a bidirectional process. Based on the estimated distributions of the terms in Eq. (32), we conducted 10⁵ *Monte Carlo* simulations to generate possible *Q* values. Fig. 5 presents the distributions of each term, while Subsections 3.1.1 and 3.1.2 detail the methodological approach used for these simulations.

The *Q*_Fisher-Escolà_ distribution, based on 10⁵ simulations, included an estimation of its density function using the *Kernel Density Estimation* (KDE) method, whose equation is given in (35):

(35) $$\hat{f}\left(x\right)=\frac{1}{nh}\sum_{i=1}^{n}K\left(\frac{x-X_{i}}{h}\right)$$

where *K*(*x*) is a *Kernel* function and *h* is the bandwidth.

The *Monte Carlo* simulations of *Q*_Fisher-Escolà_ aimed to derive a potential function for its distribution, which was then used to perform a *Maximum Likelihood Estimation* (MLE) to model *Q*_Fisher-Escolà_ based on existing mathematical frameworks. This step required analyzing the statistical properties of *Q*_Fisher-Escolà_ and testing the goodness-of-fit hypothesis between its distribution and various models used in statistical inference. This analysis was formulated by first considering the visualization expressed in Eq. (36) of the *Q*_Fisher-Escolà_ distribution:

(36) $${\hat{f}}_{Q}\left(x\right)=f\left(x;\theta_{1},\theta_{2},\cdots \right)$$

We then formulated the hypothesis of distribution fitting, as shown in Eq. (37):

(37) $$D_{n}=\underset{x}{\sup}\left|F_{n}\left(x\right)-F\left(x\right)\right|$$

**Fig. 6:**
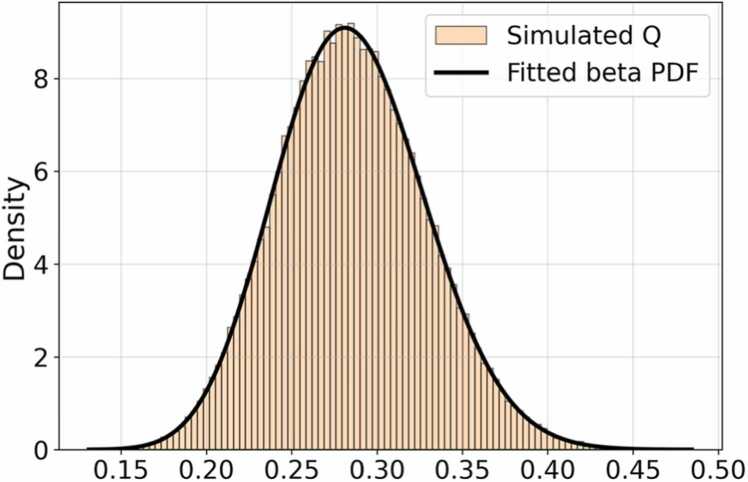
**Distribution and*****Probability Density Function*****(PDF) of*****Q***_**Fisher-Escolà**_**.** This figure illustrates the *Q*_Fisher-Escolà_ distribution based on 10^5^*Monte Carlo* random simulations, derived from the simulated distributions presented in Fig. 5. These simulations correspond to each component of the *Q*_Fisher-Escolà_ statistic (Eq. (32)).

*F*_*n*_(*x*) is the empirical distribution function and *F*(*x*) is the theoretical one. We used the *Kolmogorov-Smirnov* (KS) criterion to evaluate which model showed the least discrepancies with the simulated distribution of *Q*_Fisher-Escolà_. Fig. 6 presents the graph of the *Q*_Fisher-Escolà_ simulations along with its probability density function. The models compared to the distribution in this graph were the *normal distribution* (*p-value* = 3.4542e^-17^), *gamma distribution* (*p-value* = 0.2872), *beta distribution* (*p-value* = 0.9192), and *log-normal distribution* (*p-value* = 0.2274).

The preliminary results on the fit between distributions indicated that the *beta* modeling rules would be the most effective for estimating a new theoretical *Q*_Fisher-Escolà_ distribution. Using the MLE criterion, we obtained the parameters of the *beta distribution* based on shape properties and function boundary properties (see Eq. (38)):

(38) Beta(a,b,loc,scale)⇒Beta(21.6165,46.4970,0.0385,0.7783)

where *a* and *b* are shape parameters, while loc and scale define the function's boundaries. The descriptive statistics of the *Q*_Fisher-Escolà_ simulations showed a mean of 0.2855 and a standard deviation of 0.0436. The parameter estimates are provided in Eq. (37).

### 3.2 The theoretical distribution *Q* of Fisher-Escolà

Having obtained the *Monte Carlo* random simulations for all elements of Eq. (32) and the *Q*_Fisher-Escolà_ coefficient, as well as selecting the *beta*(α, β, a, b) statistical rule, we now distinguish two key procedures in this stage of development.

The first involves estimating the theoretical distribution of *Q*_Fisher-Escolà_, including its *Probability Density Function* (PDF) and *Cumulative Density Function* (CDF). The second focuses on formulating and computing the model’s integrals, which allow us to determine the areas under the theoretical *Q*_Fisher-Escolà_ curve. This enables the derivation of probabilities that integrate both quantum effects (*C*_*Q*_ and *I*_*Q*_) and non-quantum effects (*V*_*k*_ and β). After mathematically demonstrating these procedures, we aimed to define probabilistic decision rules for various hypothesis testing approaches, enabling the inference of potential variations transferred from quantum effects to non-quantum domains.

#### 3.2.1 Theoretical estimation by maximizing the similarity to the Q_Fisher-Escolà_ distribution

To construct a theoretical model that maximizes the properties of the *Monte Carlo* distribution of *Q*_Fisher-Escolà_ and aligns with the *beta* parameters (see Eq. (38)), we applied the MLE criterion (previously discussed) to estimate continuous values of *Q*_Fisher-Escolà_. These values define the expected theoretical model based on uncertainty principles, given that the *Monte Carlo* simulations were generated from random samples. According to our *Q*_Fisher-Escolà_ estimation logic, the resulting estimates formed a new theoretical random variable, allowing direct comparison with the results derived from Eq. (32), which serves as the *Q*_Fisher-Escolà_ test statistic. The following subsection provides a mathematical exposition of this process.

Given a distribution *Q* = {*Q*_1_, *Q*_2_, …, *Q*_*n*_}, the PDF of the *beta distribution*, parameterized as defined in Eq. (38), is formally expressed in Eq. (39):

(39) $$f\left(Q;a,b,\mathrm{loc},\mathrm{scale}\right)=\frac{{\left(Q-\mathrm{loc}\right)}^{a-1}{\left(\mathrm{scale}+\mathrm{loc}-Q\right)}^{b-1}}{{\mathrm{scale}}^{a+b-1}B\left(a,b\right)}$$

Where the *beta* function *B*(*a*, *b*) is defined in Eq. (40):

(40) $$B\left(a,b\right)=\int_{0}^{1}t^{a-1}{\left(1-t\right)}^{b-1}dt$$

and the support of *Q* is restricted to the interval (41):

(41) Q∈[loc,loc+scale]

Following this logic, the likelihood function is defined as the product of individual probabilities, expressed in Eq. (42):

(42) $$L\left(Q_{i};a,b,\mathrm{loc},\mathrm{scale}\right)=\prod_{i=1}^{n}f\left(Q_{i};a,b,\mathrm{loc},\mathrm{scale}\right)$$

We took the logarithm of the likelihood function to obtain the log-likelihood (Eq. (43)):

(43) $$\ell \left(a,b,\mathrm{loc},\mathrm{scale}\right)=\sum_{i=1}^{n}\ln\;f\left(Q_{i};a,b,\mathrm{loc},\mathrm{scale}\right)$$

expanding *f*(*Q*_*i*_) as shown in Eq. (44):

(44) $$\begin{array}{c} \ell \left(a,b,\mathrm{loc},\mathrm{scale}\right)=\\ \sum_{i=1}^{n}\left(a-1\right)\ln\left(Q_{i}-\mathrm{loc}\right)+\left(b-1\right)\times \\ \ln\left(\mathrm{scale}+\mathrm{loc}-Q_{i}\right)-\left(a+b-1\right)\ln\;\mathrm{scale}-\;\ln\;B\left(a,b\right) \end{array}$$

To find the optimal values for *a*, *b*, loc, and scale, we solve the system of equations obtained by setting the first-order partial derivatives of the log-likelihood equal to zero. The derivative with respect to *a* is given by Eq. (45):

(45) $$\frac{\partial \ell }{\partial a}=\sum_{i=1}^{n}\ln\left(Q_{i}-\mathrm{loc}\right)-n\psi \left(a\right)+n\psi \left(a+b\right)=0$$

and with respect to *b* is given by Eq. (46):

(46) $$\frac{\partial \ell }{\partial b}=\sum_{i=1}^{n}\ln\left(\mathrm{scale}+\mathrm{loc}-Q_{i}\right)-n\psi \left(b\right)+n\psi \left(a+b\right)=0$$

where ψ(*x*) is the digamma function (Eq. (47)):

(47) $$\psi \left(x\right)=\frac{d}{dx}\;\ln\;\Gamma \left(x\right)=\frac{\Gamma \prime \left(x\right)}{\Gamma \left(x\right)}$$

The derivative with respect to scale is expressed in Eq. (48):

(48) $$\frac{\partial \ell }{\partial \mathrm{scale}}=-\frac{n\left(a+b-1\right)}{\mathrm{scale}}+\left(b-1\right)\sum_{i=1}^{n}\frac{1}{\mathrm{scale}+\mathrm{loc}-Q_{i}}=0$$

and with respect to loc is given by Eq. (49):

(49) $$\frac{\partial \ell }{\partial \mathrm{loc}}=\left(a-1\right)\sum_{i=1}^{n}\frac{1}{Q_{i}-\mathrm{loc}}-\left(b-1\right)\sum_{i=1}^{n}\frac{1}{\mathrm{scale}+\mathrm{loc}-Q_{i}}=0$$

At this stage, it is essential to recognize that the previous equations do not have a closed-form analytical solution. Therefore, we employ iterative numerical methods based on the *Newton-Raphson* algorithm, also known as gradient-based numerical methods. The first step in this approach is to estimate the *beta* parameters using the method of moments (50):

(50) $$\begin{array}{c} \hat{a}=\frac{\bar{Q}\left(1-\bar{Q}\right)}{s^{2}}-1\\ \hat{b}=\frac{\left(1-\bar{Q}\right)}{\bar{Q}}\hat{a} \end{array}$$

Next, iterative steps are applied to update the values of *a*, *b*, loc, and scale through numerical optimization of the log-likelihood, typically using Eq. (51):

(51) $$\theta^{\left(t+1\right)}=\theta^{\left(t\right)}-H^{-1}\nabla \ell \left(\theta \right)$$

where *H* is the *Hessian* matrix of the second derivatives.

From this point, the algorithm iterates until ∣θ^(*t*+1)^−θ^(*t*)^∣)| is less than a tolerance threshold (ϵ).

After defining (39), (40), and (41), the PDF and CDF of the *beta distribution* were computed using (52), (53).

(52) $$\mathrm{PDF}\Rightarrow f\left(Q\right)=\frac{{\left(Q-\mathrm{loc}\right)}^{a-1}{\left(\mathrm{scale}+\mathrm{loc}-Q\right)}^{b-1}}{{\mathrm{scale}}^{a+b-1}B\left(a,b\right)}$$

and

(53) $$\mathrm{CDF}\Rightarrow F\left(Q\right)=\int_{\mathrm{loc}}^{Q}f\left(t\right)dt$$

**Fig. 7:**
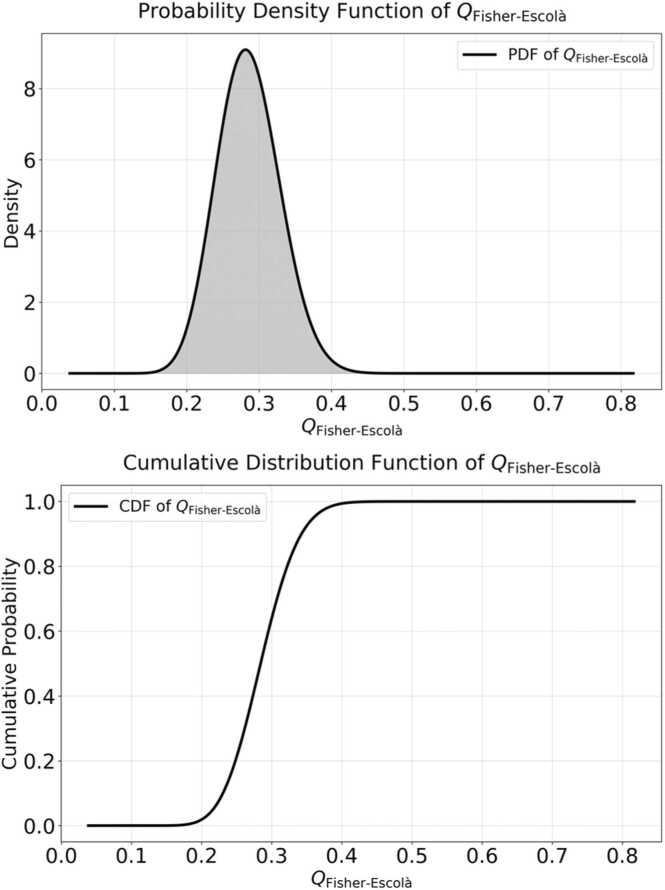
**Theoretical*****Q***_**Fisher-Escolà**_**distribution.** The theoretical *Q*_Fisher-Escolà_ distribution serves as a model for statistical inference on variances that integrate both quantum and classical probabilities. (52), (53) define the mathematical expressions and algorithms underlying these graphs.

*F*(*Q*) represents the cumulative probability up to a given value of *Q*_Fisher-Escolà_. The illustration of these functions is presented in Fig. 7. The first figure represents the distinctive element of the theoretical *Q*_Fisher-Escolà_ distribution structure.

#### 3.2.2 Development of the quantile function for critical values

If we examine Fig. 7, we observe that the second graph contains an inverse function, which served as the basis for defining the mathematical criterion to derive the quantile function for the critical values (*Q*_α_, in its most general form) of *Q*_Fisher-Escolà_. It is important to note that the quantile function varies depending on the type of hypothesis test (right-tailed test, left-tailed test, or two-tailed test). The following mathematical formulation applies to a right-tailed test, but the same logic can be used to obtain the integrals required to compute the critical values for other types of hypothesis tests. The quantile function is derived from the inverse regularized incomplete *beta* function, denoted as (54):

(54) $$I_{Q_{1-\alpha }}^{-1}\left(a,b\right)$$

and we can express *Q*_α_ as Eq. (55):

(55) $$Q_{1-\alpha }=\mathrm{loc}+\mathrm{scale}\cdot I_{1-\alpha }^{-1}\left(a,b\right)$$

where:

(56) $$I_{1-\alpha }^{-1}\left(a,b\right)=I_{Q_{1-\alpha }}\left(a,b\right)=\frac{B_{Q_{1-\alpha }}\left(a,b\right)}{B\left(a,b\right)}$$

Eq. (56) is the inverse of the regularized incomplete *beta* function, and Eq. (57) refers to the incomplete *beta* function:

(57) $$B_{Q_{1-\alpha }}\left(a,b\right)={\int_{0}^{Q_{1-\alpha }}t^{a-1}\left(1-t\right)}^{b-1}dt$$

and Eq. (58) is the *beta* complete function:

(58) $$B\left(a,b\right)=\int_{0}^{1}t^{a-1}{\left(1-t\right)}^{b-1}dt$$

There are numerical solutions for Eq. (56), which represents the quantile of the standard *beta distribution* in the interval [0,1]. Eq. (59) simplifies the logic of the quantile function for a right-tailed test:

(59) $$F\left(Q_{1-\alpha }\right)=\frac{\int_{0}^{Q_{1-\alpha }}t^{a-1}{\left(1-t\right)}^{b-1}dt}{\int_{0}^{1}t^{a-1}{\left(1-t\right)}^{b-1}dt}=I_{Q_{1-\alpha }}\left(a,b\right)=I_{1-\alpha }^{-1}\left(a,b\right)$$

(60), (61) were used for the left tailed and two-tailed tests:

(60) $$F\left(Q_{\alpha }\right)=\frac{\int_{0}^{Q_{\alpha }}t^{a-1}{\left(1-t\right)}^{b-1}dt}{\int_{0}^{1}t^{a-1}{\left(1-t\right)}^{b-1}dt}=I_{Q_{\alpha }}\left(a,b\right)=I_{\alpha }^{-1}\left(a,b\right)$$

and

(61) $$\begin{array}{l} F\left(Q_{\alpha /2}\right)=\frac{\int_{0}^{Q_{\alpha /2}}t^{a-1}{\left(1-t\right)}^{b-1}dt}{\int_{0}^{1}t^{a-1}{\left(1-t\right)}^{b-1}dt}=I_{Q_{\alpha /2}}\left(a,b\right)=I_{\alpha /2}^{-1}\left(a,b\right)\\ F\left(Q_{1-\alpha /2}\right)=\frac{\int_{0}^{Q_{1-\alpha /2}}t^{a-1}{\left(1-t\right)}^{b-1}dt}{\int_{0}^{1}t^{a-1}{\left(1-t\right)}^{b-1}dt}=I_{Q_{1-\alpha /2}}\left(a,b\right)=I_{1-\alpha /2}^{-1}\left(a,b\right) \end{array}$$

The calculation of these equations and the corresponding *Q*_α_ values for significance levels α ∈ {0.05, 0.01, 0.001, 0.0001}, along with their verification through integration, are summarized in Table 1.

**Table 1:** Critical values of *Q*_Fisher-Escolà_, extracted from its theoretical distribution, for hypothesis testing.

**Significance level (α)**	***Q***_**1-α**_**for right tailed test**	***Q***_**α**_**for left tailed test**	**For two-tailed test**	
			***Q***_**α/2**_**lower**	***Q***_**1-α/2**_**upper**
0.0500	0.3596	0.2162	0.2044	0.3745
0.0100	0.3920	0.1911	0.1825	0.4040
0.0010	0.4286	0.1655	0.1592	0.4382
0.0001	0.4586	0.1464	0.1415	0.4667

**Table 2:** Types of hypotheses, tests, and decision rules based on the critical value thresholds of *Q*_Fisher-Escolà_, given a selected significance level.

**Type of test**	**Decision rule**	**Decision on the null hypothesis**
Right tailed test	*Q*_Fisher-Escolà_ ≤ *Q*_1-α_	Fail to reject
	*Q*_Fisher-Escolà_ > *Q*_1-α_	Reject
Left tailed test	*Q*_Fisher-Escolà_ ≥ *Q*_α_	Fail to reject
	*Q*_Fisher-Escolà_ < *Q*_α_	Reject
Two-tailed test	*Q*_α/2_ ≤ *Q*_Fisher-Escolà_ ≤ *Q*_1-α/2_	Fail to reject
	*Q*_Fisher-Escolà_ > *Q*_1-α/2_ or *Q*_Fisher-Escolà_ < *Q*_α/2_	Reject

The thresholds in Table 1 allow us to test hypotheses regarding whether the explained variance obtained through quantum and non-quantum sources falls within the limits of the most probable space (confidence region for the null hypothesis), or whether, conversely, the observed value of *Q*_Fisher-Escolà_ exhibits significant variations that cannot be predicted by random fluctuations.

#### 3.2.3 Areas under curve

Beyond parametric hypothesis testing—which infers theoretical or population parameters—the theoretical distribution of *Q*_Fisher-Escolà_ can also be used to directly estimate probabilities for obtaining specific percentage variances. In other words, rather than solely assessing whether fluctuations in the *Q*_Fisher-Escolà_ statistic are statistically significant, we also sought to streamline the computation of integration processes using (60), (61). These methods enable the calculation of areas under the curve to determine the likelihood of a given *Q*_Fisher-Escolà_ result. This information is detailed in the integration tables in Appendix A, which include nine tables presenting areas under the curve based on a right-tailed one-sided test—the most commonly applied approach.

### 3.3 Mathematical formal demonstrations

We can start our demonstration from Eq. (32), where *V*_*k*_∼*F*(68, 37), β∼*F*(104, 1), *C*_*Q*_∼*F*(149, 9), and *I*_*Q*_∼*N*(μ, σ^2^). Our goal is to verify whether the distribution of *Q*_Fisher-Escolà_ follows the functional form of the *beta distribution* (62):

(62) $$f(Q)=\frac{Q^{a-1}{\left(1-Q\right)}^{b-1}}{B(a,b)},0<Q<1$$

where is the *B*(*a*, *b*) function for normalization.

*X* is a product of random variables β, *C*_*Q*_, *I*_*Q*_ and *V*_*k*_, and acts as a scaling factor. Then, the joint density function of *Q*_Fisher-Escolà_ as an integral over the densities of the individual components is shown in Eq. (63):

(63) $$f_{Q}(Q)=\int_{0}^{1}\int_{0}^{1}\int_{0}^{1}\int_{-\infty }^{\infty }f_{V_{k}}(v)f_{\beta }(\beta )f_{C_{q}}(c_{q})f_{I_{q}}(i_{q})\left|\frac{dV_{k}}{dQ}\right|dvd\beta dc_{q}di_{q}$$

where the individual densities are expressed in (64), (65), (66), (67):

(64) $$f_{V_{k}}(v)=\frac{{\left(d_{1}/d_{2}\right)}^{d_{1}/2}v^{(d_{1}/2)-1}}{B(d_{1}/2,d_{2}/2){\left(1+d_{1}v/d_{2}\right)}^{(d_{1}+d_{2})/2}}$$

(65) $$f_{\beta }(\beta )=\frac{{\left(d_{3}/d_{4}\right)}^{d_{3}/2}\beta^{(d_{3}/2)-1}}{B(d_{3}/2,d_{4}/2){\left(1+d_{3}\beta /d_{4}\right)}^{(d_{3}+d_{4})/2}}$$

(66) $$f_{C_{q}}(c_{q})=\frac{{\left(d_{5}/d_{6}\right)}^{d_{5}/2}c_{q}^{(d_{5}/2)-1}}{B(d_{5}/2,d_{6}/2){\left(1+d_{5}c_{q}/d_{6}\right)}^{(d_{5}+d_{6})/2}}$$

and

(67) $$f_{I_{q}}(i_{q})=\frac{1}{\sqrt{2{\pi \sigma }^{2}}}e^{-{\left(i_{q}-\mu \right)}^{2}/(2\sigma^{2})}$$

From this point, we use the transformation (Eq. (68)):

(68) $$X=1+\beta C_{q}(4I_{q})$$

and rewrite it as Eq. (69):

(69) $$Q_{\mathrm{Fisher}-\mathrm{Escol}à}=V_{k}\cdot X$$

Applying the change of variables and simplifying, we derive process (70):

(70) $$\begin{array}{l} f_{Q}(Q)=\int_{0}^{1}\int_{0}^{1}\int_{0}^{1}\int_{-\infty }^{\infty }\frac{{\left(d_{1}/d_{2}\right)}^{d_{1}/2}v^{(d_{1}/2)-1}}{B(d_{1}/2,d_{2}/2){\left(1+d_{1}v/d_{2}\right)}^{(d_{1}+d_{2})/2}}\times \\ \frac{{\left(d_{3}/d_{4}\right)}^{d_{3}/2}\beta^{(d_{3}/2)-1}}{B(d_{3}/2,d_{4}/2){\left(1+d_{3}\beta /d_{4}\right)}^{(d_{3}+d_{4})/2}}\times \\ \frac{{\left(d_{5}/d_{6}\right)}^{d_{5}/2}c_{q}^{(d_{5}/2)-1}}{B(d_{5}/2,d_{6}/2){\left(1+d_{5}c_{q}/d_{6}\right)}^{(d_{5}+d_{6})/2}}\times \\ \frac{1}{\sqrt{2{\pi \sigma }^{2}}}e^{-{\left(i_{q}-\mu \right)}^{2}/(2\sigma^{2})}\left|\frac{dV_{k}}{dQ}\right|dv\;d\beta \;dc_{q}\;di_{q} \end{array}$$

After solving the integral and normalizing, we obtain the final form, shown as Eq. (71):

(71) $$f_{Q}(Q)\approx cQ^{a-1}{\left(1-Q\right)}^{b-1}$$

where *c* is a normalization constant ensuring that the total probability integrates to 1. This confirms that *Q*_Fisher-Escolà_ fits a *beta distribution* under the given assumptions. Therefore, we have mathematically demonstrated the property that ensures *Q*_Fisher-Escolà_ follows a *beta distribution* by applying transformations, integrating the joint density function, and verifying the final functional form.

### 3.4 Efficacy: Type I and II errors analysis

Having mathematically and formally demonstrated that the *Q*_Fisher-Escolà_ statistic follows a *beta distribution* fitted to the parameters of Eq. (38), it is essential to assess the extent to which this new model enables successful statistical decision-making. This leads to an evaluation of the potential for inferential judgment errors when applying hypothesis testing based on the *Q*_Fisher-Escolà_ distribution. To explore this, we conducted 10^5^ *Monte Carlo* simulations of the *Q*_Fisher-Escolà_ statistic under three distinct scenarios. In the first scenario, low thresholds were set for *Q*_Fisher-Escolà_ values (ranging from 0.122 to 0.284); in the second, *Q*_Fisher-Escolà_ values were high (ranging from 0.284 to 0.485); and in the third, the statistic’s values were fully varied, covering both low and high ranges (0.119–0.490). The purpose of generating these three scenarios was to assess the robustness of the theoretical *Q*_Fisher-Escolà_ distribution under different conditions. Readers familiar with statistics will recognize that the first scenario establishes conservative conditions favoring the retention of the null hypothesis, the second introduces highly permissive conditions for rejecting it, and the third represents the most realistic case, as it includes the full range of observed *Q*_Fisher-Escolà_ values.

If our reasoning was correct and the theoretical distribution proved effective, Type I error rates (false positives) should be zero in right-tailed one-sided tests under scenario 1. In such tests, scenario 2 should yield slightly higher values, while in scenario 3, all values should remain below their corresponding significance levels. For left-tailed one-sided tests, Type II error rates (false negatives) were expected to be zero in scenario 2, whereas scenario 1 might show an increase in false negatives. In scenario 3, error rates should align with their respective significance levels. Finally, in two-tailed tests, if the distribution was truly reliable and demonstrated sufficient statistical power, both error types should remain within acceptable thresholds.

The *Monte Carlo* simulations were conducted using the estimated parameters of the *beta distribution* rule. If the reader questions whether this approach is appropriate, our response is unequivocally affirmative. This is because the initial simulations and estimations of the terms in the *Q*_Fisher-Escolà_ equation were not based on the *beta* function and distribution rules; rather, they followed *Fisher’s* and *Gaussian* principles. At this stage of the process, we were effectively undertaking a deductive *return phase.*

If the *beta distribution* is indeed the correct model governing variations in *Q*_Fisher-Escolà_, then the parameters used in these simulations should be *beta*-derived. Conversely, if the *beta* rule were not the mathematical foundation of the *Q*_Fisher-Escolà_ distribution, we would expect to observe Type I and Type II error rates across the three specified scenarios. This information is detailed in Table 3, Table 4, Table 5.

**Table 3:** Assessment of **false positive rates** (Type I errors) in **right-tailed** hypothesis testing with the *Q*_Fisher-Escolà_ Statistic.

Levels of α	Scenario 1: conservative (low values of *Q*_Fisher-Escolà_)	Scenario 2: non-conservative (high values of *Q*_Fisher-Escolà_)	Scenario 3: mixed values of *Q*_Fisher-Escolà_	Expected rates of Type I errors
0.05	∼0	0.099	0.049	5 %
0.01	∼0	0.019	0.009	1 %
0.001	∼0	0.002	0.001	0.1 %
0.0001	∼0	0.0001	∼0	0.01 %

**Table 4:** Assessment of **false negative rates** (Type II errors) in **left-tailed** hypothesis testing with the *Q*_Fisher-Escolà_ Statistic.

Levels of α	Scenario 1: conservative (low values of *Q*_Fisher-Escolà_)	Scenario 2: non-conservative (high values of *Q*_Fisher-Escolà_)	Scenario 3: mixed values of *Q*_Fisher-Escolà_	Expected rates of Type II errors
0.05	0.101	∼0	0.050	5 %
0.01	0.019	∼0	0.009	1 %
0.001	0.001	∼0	0.001	0.1 %
0.0001	∼0	∼0	∼0	0.01 %

**Table 5:** Assessment of **combined false negative and positive rates** (Type I and II errors) in **two-tailed** hypothesis testing with the *Q*_Fisher-Escolà_ Statistic.

Levels of α	Scenario 1: conservative (low values of *Q*_Fisher-Escolà_)	Scenario 2: non-conservative (high values of *Q*_Fisher-Escolà_)	Scenario 3: mixed values of *Q*_Fisher-Escolà_	Expected rates of Type I and II errors
0.05	0.050	0.050	0.049	5 %
0.01	0.010	0.010	0.009	1 %
0.001	0.001	0.001	∼0	0.1 %
0.0001	∼0	∼0	∼0	0.01 %

The results from Table 3, Table 4, Table 5 are consistent with the theoretical deductions outlined at the beginning of this subsection. Type I error rates were slightly higher in the right-tailed one-sided test for the scenario with the highest *Q*_Fisher-Escolà_ values (scenario 2). Conversely, when the statistic values were lower, the probability of Type II errors increased. In two-tailed tests, error rates remained within the assigned significance levels (corresponding to the row values). These findings confirm that the *Q* of Fisher-Escolà theoretical distribution is a reliable and effective tool for statistical inference in hypothesis testing that integrates both quantum and classical probabilities.

## 4 Discussion

The purpose of this research was to optimize the *Q* statistic for applicability in multipartite quantum systems used in configuring exposure and stimulus contingencies in implicit learning processes, while also introducing a new theoretical distribution that enables the statistical representation and inference of variations in the *Q*_Fisher-Escolà_.

The necessity and relevance of developing this model were supported by the fact that the *Q* and *Q*_Fisher-Escolà_ statistics provide probabilities or proportions of quantum variability (violating *Bell*’s or *Mermin*’s inequality) and variability related to latent factors, which could explain patterns in the response matrix structures (denoted as *V*_*k*_ in both versions of the *Q* and *Q*_Fisher-Escolà_ equations).

Although our analyses and results successfully demonstrated the mathematical foundation, effectiveness, and functionality of the *Q* of Fisher-Escolà distribution, we aim to focus the discussion on several key aspects: how to apply this new distribution, what further analyses and refinements could be made in future studies, and its potential utility in researching the phenomenology of conscious experiences. This reflection seeks to connect our new distribution and its applications with theoretical models of consciousness that conceptualize it as part of a quantum-driven mechanism occurring across multiple domains—from molecular biology and cognitive science to computational models, ultimately influencing learning processes through the entangled configuration of stimulus exposure in implicit learning sequences.

### 4.1 Basic statistical assumptions

Theoretical probability models in statistics are not universally applicable and require predefined assumptions and conditions that, while not always directly verifiable, are generally accepted as valid. This subsection outlines the theoretical and statistical conditions necessary for applying the *Q* of Fisher-Escolà distribution.

#### 4.1.1 Quantum domain requirements

The quantum requirements are grounded in well-established analyses and properties within this domain:

1) *Significant nonlocal correlations* – Ideally, nonlocal correlations in entangled systems should be greater than 0, and preferably greater than 1 [49]. While nonlocal correlations alone do not confirm entanglement, they require density matrix structures that facilitate it. Therefore, this is a desirable—though not strictly essential—condition.
2) *Entanglement of system states* – The *C*_*Q*_ and *I*_*Q*_ terms in Eq. (32) were derived from 150 density matrices of a system generating entangled qubit states. Given the intended application of this new distribution, performing calculations on *C*_*Q*_ and *I*_*Q*_ using density matrices that do not ensure entanglement would be meaningless. Thus, a minimum threshold of *C*_*Q*_ > 0.5 must be met, as proposed by Wootters [73] as an indicator that quantum objects were entangled before collapse. Ideally, *C*_*Q*_ > 0.8 is preferred, though values above 0.5 may still be informative regarding entanglement states.
3) *Violation of local statistical limit inequalities* – As demonstrated in the supplementary material (see Density_matrices_results), all density matrices in our circuit clearly violated *Mermin*’s inequality, confirming that the qubits were entangled. Without violating local statistical limits, quantum entanglement would be mathematically impossible, and the phasic behavior of qubits would not transition in a way that affects their collapse. This condition is essential and can be statistically validated [25].
4) *Entropy levels* – A final quantum factor to consider is von Neumann entropy [72]. To ensure pure entangled states, the entropy of the density matrix should ideally be low or close to 0. When von Neumann entropy approaches 1, it indicates that latent variables cannot predict the density matrix values. However, in pure entanglement, entropy should be near zero, ensuring that entanglement alone determines the qubit states. While high entropy (∼1) is not problematic in mixed or partially entangled states, pure conditions require values closer to zero.

#### 4.1.2 Non-quantum domain requirements

For the non-quantum terms in the equation, the expected linear properties should be met:

1) The explained variance (*V*_*k*_) of the response matrix, if extracted via factor analysis (as in [12]), should ideally be based on large sample sizes (preferably over 100 observations). If the sample size is small, factor analysis is still applicable, but variance proportions above 20 % should not be expected, as this is a common threshold in such scenarios. Sample size impacts pattern detection, following the *Central Limit Theorem* [10]. This is particularly relevant in our context, as it also suggests that responses to each experimental trial should follow a *Gaussian* distribution [61]. If normality is not met, robust factorization techniques can be applied [51].
2) The weight of β has been previously discussed in Escolà-Gascón [12], where several estimation methods were proposed. To ensure that the *Q* distribution is not purely theoretical, it should be grounded in the explained variance of the coefficients detailed in subsection 3.1.1, which derive from Fisher’s rule and are mathematically demonstrated in the same section.
3) If all terms of Eq. (32) are available except for β, it can be estimated by minimizing the difference between the observed and theoretical *Q*_Fisher-Escolà_ values. While this error-minimization approach could serve as an alternative, we recommend obtaining β from experimentally validated and empirically tested effects.

### 4.2 Applications and safeguards against misuse

The first point we wish to emphasize is an important yet easily overlooked fact: constructing a new theoretical distribution that allows us to compute probabilities for explained variance—integrating quantum and non-quantum effects in learning sequences—does not imply or demonstrate that conscious experience has a quantum origin. This study presents a new statistical research tool, specifically designed for analyzing phase transition effects in qubit states, which we propose for configuring stimulus exposure and contingencies. While these effects play a role in the phenomenology of learning processes, they neither confirm nor disprove that consciousness itself operates on quantum principles. However, we have provided solid mathematical and statistical evidence supporting the idea that certain aspects of consciousness may align with quantum mechanisms. While still preliminary, this evidence is promising for two reasons:

1) It introduces *empirical testability* into several quantum theories cited in the introduction [35], making them amenable to real-world validation. This capability is what makes the *Q* of Fisher-Escolà theoretical distribution a potentially groundbreaking tool in this field. As a reminder, no theory can hold scientific credibility without empirical-statistical validation [36]. Facilitating statistical methodologies that transition theories into applicable frameworks is a critical requirement of scientific research—one that defines the primary significance of this new distribution.
2) What we have demonstrated regarding the *Q* of Fisher-Escolà distribution does not resolve the decoherence problem described by Tegmark [65]. However, it provides a mathematically functional approach for conducting statistical tests without decoherence obstructing further experimentation in this direction. This is a key application that must be highlighted to prevent potential misinterpretations. We have not solved decoherence, as it arises logically when sufficient variability is introduced into a matrix, making it susceptible to classical linear statistical analysis. This issue is formally captured in the *Fisher-Escolà paradox*: stabilizing the integral of the QFI by dividing it by the maximum density function value serves as a functional correction but does not constitute a structural solution to the problem. Therefore, we anticipate that future studies using the *Q* of Fisher-Escolà distribution may still encounter decoherence-related limitations. To mitigate this issue, we recommend using the EM1 system-circuit, as implemented in this study. Although it is not a perfect circuit, it demonstrated consistent effectiveness across 150 distinct density matrices, meeting the required quantum properties—a notable result that supports its adoption by other researchers.

A final limitation concerns the development process of the *Q* of Fisher-Escolà distribution. While basing our approach on formal logical criteria for modeling the distribution of values in Eq. (32) is a valid choice, it does not exclude alternative methodological approaches. In this regard, we emphasize that this comprehensive study represents only an initial framework for implementing hypothesis testing in the transition of quantum effects to non-quantum domains. We see this as a significant step forward in expanding research possibilities on consciousness and quantum cognition.

Our findings are not intended as final or exclusive, and we encourage fellow researchers to contribute to advancing and refining this line of inquiry. Rather than presenting the final frontier, we believe this work marks the first scientific frontier in exploring the limits of our current understanding.

### 4.3 Clinical and translational perspectives: toward quantum-integrated neuroscience

While the present work is fundamentally methodological and mathematical, the implications of the Fisher-Escolà *Q* distribution may extend into several clinically relevant domains within neuroscience and biomedical research. The capacity of the *Q*_Fisher-Escolà_ model to quantify explained variance arising from both classical and quantum informational sources enables novel inferential strategies that could, in future applications, inform diagnostics and personalized therapeutics. For example, neurodegenerative diseases such as *Alzheimer's disease* (AD) and *Parkinson's disease* (PD) are characterized by subtle and often preclinical shifts in cognitive performance and neural connectivity [4]. These early changes may involve nonlinear and nonlocal disruptions that are poorly captured by conventional statistical approaches. Recent studies have demonstrated that quantum-inspired machine learning models can successfully detect early-stage AD and PD with high accuracy, integrating multimodal biomarkers and capturing complex feature interactions that escape classical frameworks [30], [68].

In neuropsychiatry and consciousness research, the Fisher-Escolà *Q* distribution may offer a unique framework for modeling hybrid variance arising from both observable and nonlocal influences. Recent experimental work suggests that quantum entanglement effects, implemented through bipartite qubit circuits, may modulate behavioral responses in implicit learning paradigms [12]. In contrast, parallel studies employing electroencephalography-based quantum potential metrics have demonstrated high diagnostic utility in major depressive disorder, schizophrenia, and cognitive decline [62]. Furthermore, theoretical neuroscience frameworks increasingly recognize the potential coupling of brain electromagnetic activity with endogenous quantum fields, supporting the hypothesis that quantum coherence or decoherence may play roles in altered states of consciousness and neuropsychiatric dysfunction [29].

Finally, the translational impact of quantum-classical inference models may extend to adaptive closed-loop *brain-computer interface* (BCI) systems [28]. BCIs capable of dynamically adjusting feedback based on internal state estimation could benefit from metrics sensitive to entanglement, coherence, or Fisher information in neural signals. Quantum-enhanced BCI architectures are already being explored for their potential to improve decision-making speed, error correction, and signal classification robustness. In this context, the Fisher-Escolà *Q* distribution may function as an integrative decision criterion within such interfaces, regulating stimulus-response contingencies by quantifying latent cognitive dynamics inaccessible through classical signal decoding alone. While speculative, these convergences between quantum modeling and clinical neuroscience open promising interdisciplinary avenues that warrant future empirical validation.

### 4.4 Conclusions of the present study

This report presents the statistical and mathematical discoveries that emerged from quantum mathematics-based theories aimed at explaining and predicting subjective conscious experiences. Inspired by the experimental work of Escolà-Gascón [12] and the principles of geneticist-engineer Ronald A. Fisher—with 2025 marking the centennial of the latter’s contributions [17]—we propose a new theoretical distribution, the *Q* of Fisher-Escolà, designed to model phenomena arising from the fusion of quantum and non-quantum ontological effects.

While our findings present significant challenges, they allow us to conclude that:

1) The *Q* of Fisher-Escolà distribution is a statistically robust tool for inference in this context.
2) The analytical solution to the QFI quantum integral successfully stabilizes decoherence, ensuring balanced information levels—neither excessively sensitive to contextual perturbations (which induce decoherence) nor overly restrictive, thereby preserving quantum information processing across the 150 systems analyzed through 150 quantum and empirical density matrices.
3) The *Q* of Fisher-Escolà distribution demonstrates no Type I or Type II errors when applied in bilateral hypothesis testing. The rates of false positives and false negatives were negligible, supporting its classification as a high-power distribution with potential external validity, particularly when used under probabilistic sampling conditions.
4) This new distribution opens new avenues for testing consciousness theories, particularly those suggesting that quantum entanglement-based effects play a role in the emergence of conscious experience.
5) Ultimately, the *Q* of Fisher-Escolà distribution provides a novel statistical framework at the intersection of quantum mechanics and consciousness research.

Moving forward, the key challenges for this new Fisher-Escolà statistical model will lie in its experimental applications and in refining its mathematical properties to further enhance its probabilistic accuracy. While the logic of hypothesis testing is well established, the mathematical framework introduced here is entirely new. In the face of emerging quantum challenges, scientific simplicity and functional design become especially valuable—both for ensuring experimental reproducibility and for broadening practical applicability. This is precisely what the *Q* of Fisher-Escolà distribution offers at the intersection of quantum mechanics and consciousness research.

## Funding statement

This research was supported, through *Prof. Dr. Julián Benito-León*, by the *National Institutes of Health* (NINDS #R01 NS39422 and R01 NS094607) and by the Recovery, Transformation, and Resilience Plan of the *Spanish Ministry of Science and Innovation* (grants TED2021-130174B-C33, NETremor, and PID2022-138585OB-C33, Resonate). This publication was funded by project TED2021-130174B-C33, supported by MCIN/AEI/10.13039/501100011033 and the *European Union's "NextGenerationEU"/PRTR*.

## Authors statement

This research was conducted entirely by *Prof. Dr. Álex Escolà-Gascón* in collaboration with *Prof. Dr. Julián Benito-León*.

## Ethical statement

The Committee for Ethical Guarantees of the Present University conducted a favorable review of the research protocol. In the original experiment, each participant provided informed consent, which elucidated the study's objectives and the assessment tests employed. Participation was strictly voluntary, with participants retaining the right to withdraw from the study at their discretion. Additionally, all collected data were processed with utmost confidentiality and anonymity.

## CRediT authorship contribution statement

**Benito-León Julián:** Writing – review & editing, Validation, Supervision, Resources, Project administration, Investigation, Funding acquisition. **Escolà-Gascón Álex:** Writing – review & editing, Writing – original draft, Visualization, Validation, Software, Methodology, Investigation, Formal analysis, Data curation, Conceptualization.

## Declaration of Competing Interest

The authors wish to confirm that there are no known conflicts of interest associated with this publication.
