# Supplementary material for: Mathematical proof of the Fisher-Escolà Q statistical distribution in quantum consciousness modeling
Source: Comput Struct Biotechnol J. 2025 Apr 26;30:41–58. doi: 10.1016/j.csbj.2025.04.025 (PMC12137171; doi:10.1016/j.csbj.2025.04.025)
Supplement: Supplementary file 4 — Supplementary material [file mmc4.pdf]

Appendix A: Table of Integrals

Q distribution of Fisher-Escolà for testing quantum probability transitions. Prepared by *Prof. Dr. Álex Escolà-Gascón*

Third and fourth decimal places

$Q_{\text{Fisher-Escolà}}$	0.0000	0.0001	0.0002	0.0003	0.0004	0.0005	0.0006	0.0007	0.0008	0.0009	0.0010	0.0011
**0.1300**	~0	~0	~0	~0	~0	~0	~0	~0	~0	~0	~0	~0
**0.1400**	~0	~0	~0	~0	~0	~0	~0	~0	~0	~0	~0	~0
**0.1500**	0.0002	0.0002	0.0002	0.0002	0.0002	0.0002	0.0002	0.0002	0.0002	0.0002	0.0002	0.0002
**0.1600**	0.0005	0.0006	0.0006	0.0006	0.0006	0.0006	0.0006	0.0006	0.0006	0.0006	0.0006	0.0006
**0.1700**	0.0016	0.0016	0.0016	0.0016	0.0017	0.0017	0.0017	0.0017	0.0017	0.0017	0.0018	0.0018
**0.1800**	0.0040	0.0041	0.0041	0.0042	0.0042	0.0042	0.0043	0.0043	0.0043	0.0044	0.0044	0.0044
**0.1900**	0.0092	0.0092	0.0093	0.0094	0.0095	0.0095	0.0096	0.0097	0.0097	0.0098	0.0099	0.0100
**0.2000**	0.0188	0.0189	0.0191	0.0192	0.0193	0.0194	0.0196	0.0197	0.0198	0.0200	0.0201	0.0202
**0.2100**	0.0352	0.0355	0.0357	0.0359	0.0361	0.0363	0.0365	0.0367	0.0369	0.0371	0.0374	0.0376
**0.2200**	0.0610	0.0613	0.0617	0.0620	0.0623	0.0626	0.0629	0.0633	0.0636	0.0639	0.0642	0.0645
**0.2300**	0.0984	0.0989	0.0993	0.0998	0.1002	0.1006	0.1011	0.1015	0.1020	0.1024	0.1029	0.1033
**0.2400**	0.1489	0.1495	0.1500	0.1506	0.1512	0.1518	0.1524	0.1529	0.1535	0.1541	0.1547	0.1553
**0.2500**	0.2126	0.2133	0.2140	0.2147	0.2154	0.2162	0.2169	0.2176	0.2183	0.2190	0.2197	0.2204
**0.2600**	0.2883	0.2891	0.2899	0.2907	0.2915	0.2923	0.2932	0.2940	0.2948	0.2956	0.2964	0.2972
**0.2700**	0.3730	0.3739	0.3748	0.3757	0.3765	0.3774	0.3783	0.3792	0.3801	0.3810	0.3818	0.3827
**0.2800**	0.4628	0.4638	0.4647	0.4656	0.4665	0.4674	0.4683	0.4692	0.4701	0.4710	0.4719	0.4728
**0.2900**	0.5532	0.5541	0.5550	0.5559	0.5568	0.5577	0.5586	0.5595	0.5604	0.5612	0.5621	0.5630
**0.3000**	0.6398	0.6406	0.6414	0.6423	0.6431	0.6439	0.6447	0.6456	0.6464	0.6472	0.6480	0.6489
**0.3100**	0.7187	0.7195	0.7202	0.7209	0.7217	0.7224	0.7232	0.7239	0.7246	0.7254	0.7261	0.7268
**0.3200**	0.7875	0.7881	0.7888	0.7894	0.7900	0.7907	0.7913	0.7919	0.7925	0.7932	0.7938	0.7944
**0.3300**	0.8448	0.8453	0.8459	0.8464	0.8469	0.8474	0.8479	0.8484	0.8489	0.8494	0.8499	0.8504
**0.3400**	0.8905	0.8909	0.8913	0.8917	0.8921	0.8925	0.8929	0.8933	0.8937	0.8941	0.8945	0.8949
**0.3500**	0.9254	0.9257	0.9260	0.9263	0.9266	0.9269	0.9272	0.9275	0.9278	0.9281	0.9284	0.9287
**0.3600**	0.9510	0.9512	0.9514	0.9516	0.9518	0.9520	0.9522	0.9525	0.9527	0.9529	0.9531	0.9533
**0.3700**	0.9689	0.9691	0.9692	0.9694	0.9695	0.9696	0.9698	0.9699	0.9701	0.9702	0.9704	0.9705
**0.3800**	0.9810	0.9811	0.9812	0.9813	0.9814	0.9815	0.9816	0.9817	0.9818	0.9819	0.9820	0.9821
**0.3900**	0.9888	0.9889	0.9889	0.9890	0.9891	0.9891	0.9892	0.9892	0.9893	0.9894	0.9894	0.9895
**0.4000**	0.9937	0.9937	0.9937	0.9938	0.9938	0.9939	0.9939	0.9939	0.9940	0.9940	0.9940	0.9941
**0.4100**	0.9966	0.9966	0.9966	0.9966	0.9966	0.9967	0.9967	0.9967	0.9967	0.9967	0.9968	0.9968
**0.4200**	0.9982	0.9982	0.9982	0.9982	0.9982	0.9983	0.9983	0.9983	0.9983	0.9983	0.9983	0.9983
**0.4300**	0.9991	0.9991	0.9991	0.9991	0.9991	0.9991	0.9991	0.9991	0.9991	0.9991	0.9992	0.9992
**0.4400**	0.9996	0.9996	0.9996	0.9996	0.9996	0.9996	0.9996	0.9996	0.9996	0.9996	0.9996	0.9996
**0.4500**	0.9998	0.9998	0.9998	0.9998	0.9998	0.9998	0.9998	0.9998	0.9998	0.9998	0.9998	0.9998
**0.4600**	0.9999	0.9999	0.9999	0.9999	0.9999	0.9999	0.9999	0.9999	0.9999	0.9999	0.9999	0.9999
**0.4700**	1.0000	1.0000	1.0000	1.0000	1.0000	1.0000	1.0000	1.0000	1.0000	1.0000	1.0000	1.0000

Appendix A: Table of Integrals (continued)

Q distribution of Fisher-Escolà for testing quantum probability transitions. Prepared by *Prof. Dr. Álex Escolà-Gascón*

Third and fourth decimal places

$Q_{\text{Fisher-Escolà}}$	0.0012	0.0013	0.0014	0.0015	0.0016	0.0017	0.0018	0.0019	0.0020	0.0021	0.0022	0.0023
**0.1300**	~0	~0	~0	~0	~0	~0	~0	~0	~0	~0	~0	~0
**0.1400**	~0	~0	~0	~0	0.0001	0.0001	0.0001	0.0001	0.0001	0.0001	0.0001	0.0001
**0.1500**	0.0002	0.0002	0.0002	0.0002	0.0002	0.0002	0.0002	0.0002	0.0002	0.0002	0.0002	0.0002
**0.1600**	0.0006	0.0006	0.0006	0.0006	0.0007	0.0007	0.0007	0.0007	0.0007	0.0007	0.0007	0.0007
**0.1700**	0.0018	0.0018	0.0018	0.0018	0.0019	0.0019	0.0019	0.0019	0.0019	0.0020	0.0020	0.0020
**0.1800**	0.0045	0.0045	0.0046	0.0046	0.0046	0.0047	0.0047	0.0048	0.0048	0.0048	0.0049	0.0049
**0.1900**	0.0100	0.0101	0.0102	0.0103	0.0104	0.0104	0.0105	0.0106	0.0107	0.0107	0.0108	0.0109
**0.2000**	0.0204	0.0205	0.0206	0.0208	0.0209	0.0210	0.0212	0.0213	0.0215	0.0216	0.0217	0.0219
**0.2100**	0.0378	0.0380	0.0382	0.0385	0.0387	0.0389	0.0391	0.0394	0.0396	0.0398	0.0400	0.0403
**0.2200**	0.0649	0.0652	0.0655	0.0659	0.0662	0.0665	0.0669	0.0672	0.0675	0.0679	0.0682	0.0685
**0.2300**	0.1038	0.1042	0.1047	0.1051	0.1056	0.1061	0.1065	0.1070	0.1075	0.1079	0.1084	0.1089
**0.2400**	0.1559	0.1564	0.1570	0.1576	0.1582	0.1588	0.1594	0.1600	0.1606	0.1612	0.1618	0.1624
**0.2500**	0.2211	0.2218	0.2226	0.2233	0.2240	0.2247	0.2254	0.2262	0.2269	0.2276	0.2283	0.2291
**0.2600**	0.2980	0.2989	0.2997	0.3005	0.3013	0.3022	0.3030	0.3038	0.3046	0.3055	0.3063	0.3071
**0.2700**	0.3836	0.3845	0.3854	0.3863	0.3872	0.3881	0.3889	0.3898	0.3907	0.3916	0.3925	0.3934
**0.2800**	0.4738	0.4747	0.4756	0.4765	0.4774	0.4783	0.4792	0.4801	0.4810	0.4819	0.4828	0.4838
**0.2900**	0.5639	0.5648	0.5657	0.5666	0.5674	0.5683	0.5692	0.5701	0.5710	0.5719	0.5727	0.5736
**0.3000**	0.6497	0.6505	0.6513	0.6522	0.6530	0.6538	0.6546	0.6554	0.6563	0.6571	0.6579	0.6587
**0.3100**	0.7276	0.7283	0.7290	0.7297	0.7305	0.7312	0.7319	0.7326	0.7334	0.7341	0.7348	0.7355
**0.3200**	0.7950	0.7956	0.7962	0.7969	0.7975	0.7981	0.7987	0.7993	0.7999	0.8005	0.8011	0.8017
**0.3300**	0.8509	0.8514	0.8519	0.8524	0.8529	0.8534	0.8539	0.8544	0.8549	0.8554	0.8559	0.8563
**0.3400**	0.8953	0.8956	0.8960	0.8964	0.8968	0.8972	0.8976	0.8979	0.8983	0.8987	0.8991	0.8995
**0.3500**	0.9289	0.9292	0.9295	0.9298	0.9301	0.9304	0.9307	0.9309	0.9312	0.9315	0.9318	0.9321
**0.3600**	0.9535	0.9537	0.9539	0.9541	0.9543	0.9545	0.9547	0.9549	0.9551	0.9553	0.9555	0.9557
**0.3700**	0.9706	0.9708	0.9709	0.9711	0.9712	0.9713	0.9715	0.9716	0.9718	0.9719	0.9720	0.9722
**0.3800**	0.9821	0.9822	0.9823	0.9824	0.9825	0.9826	0.9827	0.9828	0.9829	0.9830	0.9830	0.9831
**0.3900**	0.9895	0.9896	0.9897	0.9897	0.9898	0.9898	0.9899	0.9899	0.9900	0.9900	0.9901	0.9902
**0.4000**	0.9941	0.9941	0.9942	0.9942	0.9942	0.9943	0.9943	0.9943	0.9944	0.9944	0.9944	0.9945
**0.4100**	0.9968	0.9968	0.9968	0.9969	0.9969	0.9969	0.9969	0.9969	0.9970	0.9970	0.9970	0.9970
**0.4200**	0.9983	0.9983	0.9984	0.9984	0.9984	0.9984	0.9984	0.9984	0.9984	0.9984	0.9984	0.9985
**0.4300**	0.9992	0.9992	0.9992	0.9992	0.9992	0.9992	0.9992	0.9992	0.9992	0.9992	0.9992	0.9992
**0.4400**	0.9996	0.9996	0.9996	0.9996	0.9996	0.9996	0.9996	0.9996	0.9996	0.9996	0.9996	0.9996
**0.4500**	0.9998	0.9998	0.9998	0.9998	0.9998	0.9998	0.9998	0.9998	0.9998	0.9998	0.9998	0.9998
**0.4600**	0.9999	0.9999	0.9999	0.9999	0.9999	0.9999	0.9999	0.9999	0.9999	0.9999	0.9999	0.9999
**0.4700**	1.0000	1.0000	1.0000	1.0000	1.0000	1.0000	1.0000	1.0000	1.0000	1.0000	1.0000	1.0000

Appendix A: Table of Integrals (continued)

Q distribution of Fisher-Escolà for testing quantum probability transitions. Prepared by *Prof. Dr. Álex Escolà-Gascón*

Third and fourth decimal places

$Q_{\text{Fisher-Escolà}}$	0.0024	0.0025	0.0026	0.0027	0.0028	0.0029	0.0030	0.0031	0.0032	0.0033	0.0034	0.0035
**0.1300**	~0	~0	~0	~0	~0	~0	~0	~0	~0	~0	~0	~0
**0.1400**	0.0001	0.0001	0.0001	0.0001	0.0001	0.0001	0.0001	0.0001	0.0001	0.0001	0.0001	0.0001
**0.1500**	0.0002	0.0002	0.0002	0.0002	0.0002	0.0002	0.0002	0.0002	0.0002	0.0002	0.0002	0.0003
**0.1600**	0.0007	0.0007	0.0007	0.0007	0.0007	0.0008	0.0008	0.0008	0.0008	0.0008	0.0008	0.0008
**0.1700**	0.0020	0.0020	0.0021	0.0021	0.0021	0.0021	0.0021	0.0022	0.0022	0.0022	0.0022	0.0022
**0.1800**	0.0050	0.0050	0.0051	0.0051	0.0051	0.0052	0.0052	0.0053	0.0053	0.0054	0.0054	0.0055
**0.1900**	0.0110	0.0111	0.0112	0.0112	0.0113	0.0114	0.0115	0.0116	0.0117	0.0117	0.0118	0.0119
**0.2000**	0.0220	0.0222	0.0223	0.0225	0.0226	0.0228	0.0229	0.0231	0.0232	0.0234	0.0235	0.0237
**0.2100**	0.0405	0.0407	0.0410	0.0412	0.0414	0.0417	0.0419	0.0421	0.0424	0.0426	0.0428	0.0431
**0.2200**	0.0689	0.0692	0.0696	0.0699	0.0703	0.0706	0.0709	0.0713	0.0716	0.0720	0.0724	0.0727
**0.2300**	0.1093	0.1098	0.1103	0.1107	0.1112	0.1117	0.1122	0.1126	0.1131	0.1136	0.1141	0.1146
**0.2400**	0.1630	0.1636	0.1642	0.1648	0.1654	0.1660	0.1667	0.1673	0.1679	0.1685	0.1691	0.1697
**0.2500**	0.2298	0.2305	0.2313	0.2320	0.2327	0.2334	0.2342	0.2349	0.2357	0.2364	0.2371	0.2379
**0.2600**	0.3079	0.3088	0.3096	0.3104	0.3113	0.3121	0.3129	0.3138	0.3146	0.3154	0.3163	0.3171
**0.2700**	0.3943	0.3952	0.3961	0.3970	0.3978	0.3987	0.3996	0.4005	0.4014	0.4023	0.4032	0.4041
**0.2800**	0.4847	0.4856	0.4865	0.4874	0.4883	0.4892	0.4901	0.4910	0.4919	0.4928	0.4937	0.4947
**0.2900**	0.5745	0.5754	0.5763	0.5771	0.5780	0.5789	0.5798	0.5807	0.5815	0.5824	0.5833	0.5842
**0.3000**	0.6595	0.6603	0.6611	0.6620	0.6628	0.6636	0.6644	0.6652	0.6660	0.6668	0.6676	0.6684
**0.3100**	0.7362	0.7369	0.7377	0.7384	0.7391	0.7398	0.7405	0.7412	0.7419	0.7426	0.7433	0.7441
**0.3200**	0.8023	0.8029	0.8035	0.8041	0.8047	0.8053	0.8059	0.8065	0.8071	0.8077	0.8083	0.8089
**0.3300**	0.8568	0.8573	0.8578	0.8583	0.8588	0.8593	0.8597	0.8602	0.8607	0.8612	0.8616	0.8621
**0.3400**	0.8998	0.9002	0.9006	0.9010	0.9013	0.9017	0.9021	0.9024	0.9028	0.9032	0.9035	0.9039
**0.3500**	0.9323	0.9326	0.9329	0.9332	0.9334	0.9337	0.9340	0.9343	0.9345	0.9348	0.9351	0.9353
**0.3600**	0.9559	0.9561	0.9563	0.9565	0.9567	0.9569	0.9571	0.9573	0.9575	0.9577	0.9578	0.9580
**0.3700**	0.9723	0.9724	0.9726	0.9727	0.9728	0.9730	0.9731	0.9732	0.9733	0.9735	0.9736	0.9737
**0.3800**	0.9832	0.9833	0.9834	0.9835	0.9836	0.9837	0.9837	0.9838	0.9839	0.9840	0.9841	0.9842
**0.3900**	0.9902	0.9903	0.9903	0.9904	0.9904	0.9905	0.9905	0.9906	0.9906	0.9907	0.9907	0.9908
**0.4000**	0.9945	0.9945	0.9946	0.9946	0.9946	0.9947	0.9947	0.9947	0.9948	0.9948	0.9948	0.9949
**0.4100**	0.9970	0.9971	0.9971	0.9971	0.9971	0.9971	0.9971	0.9972	0.9972	0.9972	0.9972	0.9972
**0.4200**	0.9985	0.9985	0.9985	0.9985	0.9985	0.9985	0.9985	0.9985	0.9985	0.9986	0.9986	0.9986
**0.4300**	0.9992	0.9992	0.9992	0.9993	0.9993	0.9993	0.9993	0.9993	0.9993	0.9993	0.9993	0.9993
**0.4400**	0.9996	0.9996	0.9996	0.9996	0.9996	0.9996	0.9997	0.9997	0.9997	0.9997	0.9997	0.9997
**0.4500**	0.9998	0.9998	0.9998	0.9998	0.9998	0.9998	0.9998	0.9998	0.9998	0.9998	0.9998	0.9998
**0.4600**	0.9999	0.9999	0.9999	0.9999	0.9999	0.9999	0.9999	0.9999	0.9999	0.9999	0.9999	0.9999
**0.4700**	1.0000	1.0000	1.0000	1.0000	1.0000	1.0000	1.0000	1.0000	1.0000	1.0000	1.0000	1.0000

Appendix A: Table of Integrals (continued)

Q distribution of Fisher-Escolà for testing quantum probability transitions. Prepared by *Prof. Dr. Álex Escolà-Gascón*

	Third and fourth decimal places											
$Q_{\text{Fisher-Escolà}}$	**0.0036**	**0.0037**	**0.0038**	**0.0039**	**0.0040**	**0.0041**	**0.0042**	**0.0043**	**0.0044**	**0.0045**	**0.0046**	**0.0047**
**0.1300**	~0	~0	~0	~0	~0	~0	~0	~0	~0	~0	~0	~0
**0.1400**	0.0001	0.0001	0.0001	0.0001	0.0001	0.0001	0.0001	0.0001	0.0001	0.0001	0.0001	0.0001
**0.1500**	0.0003	0.0003	0.0003	0.0003	0.0003	0.0003	0.0003	0.0003	0.0003	0.0003	0.0003	0.0003
**0.1600**	0.0008	0.0008	0.0008	0.0008	0.0009	0.0009	0.0009	0.0009	0.0009	0.0009	0.0009	0.0009
**0.1700**	0.0023	0.0023	0.0023	0.0023	0.0023	0.0024	0.0024	0.0024	0.0024	0.0025	0.0025	0.0025
**0.1800**	0.0055	0.0055	0.0056	0.0056	0.0057	0.0057	0.0058	0.0058	0.0059	0.0059	0.0060	0.0060
**0.1900**	0.0120	0.0121	0.0122	0.0123	0.0124	0.0125	0.0125	0.0126	0.0127	0.0128	0.0129	0.0130
**0.2000**	0.0238	0.0240	0.0241	0.0243	0.0244	0.0246	0.0247	0.0249	0.0250	0.0252	0.0254	0.0255
**0.2100**	0.0433	0.0436	0.0438	0.0441	0.0443	0.0446	0.0448	0.0450	0.0453	0.0455	0.0458	0.0461
**0.2200**	0.0731	0.0734	0.0738	0.0741	0.0745	0.0749	0.0752	0.0756	0.0759	0.0763	0.0767	0.0771
**0.2300**	0.1151	0.1155	0.1160	0.1165	0.1170	0.1175	0.1180	0.1185	0.1190	0.1195	0.1200	0.1205
**0.2400**	0.1704	0.1710	0.1716	0.1722	0.1728	0.1735	0.1741	0.1747	0.1754	0.1760	0.1766	0.1772
**0.2500**	0.2386	0.2394	0.2401	0.2409	0.2416	0.2423	0.2431	0.2438	0.2446	0.2453	0.2461	0.2469
**0.2600**	0.3179	0.3188	0.3196	0.3205	0.3213	0.3221	0.3230	0.3238	0.3247	0.3255	0.3264	0.3272
**0.2700**	0.4050	0.4059	0.4068	0.4077	0.4086	0.4095	0.4104	0.4113	0.4122	0.4131	0.4140	0.4149
**0.2800**	0.4956	0.4965	0.4974	0.4983	0.4992	0.5001	0.5010	0.5019	0.5028	0.5037	0.5046	0.5055
**0.2900**	0.5850	0.5859	0.5868	0.5877	0.5885	0.5894	0.5903	0.5912	0.5920	0.5929	0.5938	0.5946
**0.3000**	0.6692	0.6700	0.6708	0.6716	0.6724	0.6732	0.6740	0.6748	0.6756	0.6764	0.6772	0.6780
**0.3100**	0.7448	0.7455	0.7462	0.7469	0.7476	0.7483	0.7490	0.7497	0.7504	0.7510	0.7517	0.7524
**0.3200**	0.8095	0.8101	0.8107	0.8113	0.8119	0.8124	0.8130	0.8136	0.8142	0.8148	0.8153	0.8159
**0.3300**	0.8626	0.8631	0.8635	0.8640	0.8645	0.8649	0.8654	0.8659	0.8663	0.8668	0.8673	0.8677
**0.3400**	0.9043	0.9046	0.9050	0.9053	0.9057	0.9061	0.9064	0.9068	0.9071	0.9075	0.9078	0.9082
**0.3500**	0.9356	0.9359	0.9361	0.9364	0.9367	0.9369	0.9372	0.9375	0.9377	0.9380	0.9382	0.9385
**0.3600**	0.9582	0.9584	0.9586	0.9588	0.9590	0.9592	0.9593	0.9595	0.9597	0.9599	0.9601	0.9602
**0.3700**	0.9739	0.9740	0.9741	0.9742	0.9744	0.9745	0.9746	0.9747	0.9749	0.9750	0.9751	0.9752
**0.3800**	0.9842	0.9843	0.9844	0.9845	0.9846	0.9846	0.9847	0.9848	0.9849	0.9850	0.9850	0.9851
**0.3900**	0.9908	0.9909	0.9910	0.9910	0.9911	0.9911	0.9912	0.9912	0.9913	0.9913	0.9914	0.9914
**0.4000**	0.9949	0.9949	0.9949	0.9950	0.9950	0.9950	0.9951	0.9951	0.9951	0.9952	0.9952	0.9952
**0.4100**	0.9973	0.9973	0.9973	0.9973	0.9973	0.9973	0.9974	0.9974	0.9974	0.9974	0.9974	0.9974
**0.4200**	0.9986	0.9986	0.9986	0.9986	0.9986	0.9986	0.9986	0.9986	0.9987	0.9987	0.9987	0.9987
**0.4300**	0.9993	0.9993	0.9993	0.9993	0.9993	0.9993	0.9993	0.9993	0.9993	0.9993	0.9993	0.9994
**0.4400**	0.9997	0.9997	0.9997	0.9997	0.9997	0.9997	0.9997	0.9997	0.9997	0.9997	0.9997	0.9997
**0.4500**	0.9998	0.9999	0.9999	0.9999	0.9999	0.9999	0.9999	0.9999	0.9999	0.9999	0.9999	0.9999
**0.4600**	0.9999	0.9999	0.9999	0.9999	0.9999	0.9999	0.9999	0.9999	0.9999	0.9999	0.9999	0.9999
**0.4700**	1.0000	1.0000	1.0000	1.0000	1.0000	1.0000	1.0000	1.0000	1.0000	1.0000	1.0000	1.0000

Appendix A: Table of Integrals (continued)

Q distribution of Fisher-Escolà for testing quantum probability transitions. Prepared by *Prof. Dr. Álex Escolà-Gascón*

	Third and fourth decimal places											
$Q_{\text{Fisher-Escolà}}$	**0.0048**	**0.0049**	**0.0050**	**0.0051**	**0.0052**	**0.0053**	**0.0054**	**0.0055**	**0.0056**	**0.0057**	**0.0058**	**0.0059**
**0.1300**	~0	~0	~0	~0	~0	~0	~0	~0	~0	~0	~0	~0
**0.1400**	0.0001	0.0001	0.0001	0.0001	0.0001	0.0001	0.0001	0.0001	0.0001	0.0001	0.0001	0.0001
**0.1500**	0.0003	0.0003	0.0003	0.0003	0.0003	0.0003	0.0003	0.0003	0.0003	0.0003	0.0003	0.0003
**0.1600**	0.0009	0.0009	0.0010	0.0010	0.0010	0.0010	0.0010	0.0010	0.0010	0.0010	0.0010	0.0010
**0.1700**	0.0025	0.0026	0.0026	0.0026	0.0026	0.0026	0.0027	0.0027	0.0027	0.0027	0.0028	0.0028
**0.1800**	0.0061	0.0061	0.0062	0.0062	0.0063	0.0063	0.0064	0.0064	0.0065	0.0065	0.0066	0.0066
**0.1900**	0.0131	0.0132	0.0133	0.0134	0.0135	0.0136	0.0137	0.0138	0.0139	0.0140	0.0141	0.0142
**0.2000**	0.0257	0.0259	0.0260	0.0262	0.0263	0.0265	0.0267	0.0268	0.0270	0.0272	0.0273	0.0275
**0.2100**	0.0463	0.0466	0.0468	0.0471	0.0473	0.0476	0.0479	0.0481	0.0484	0.0486	0.0489	0.0492
**0.2200**	0.0774	0.0778	0.0782	0.0785	0.0789	0.0793	0.0797	0.0800	0.0804	0.0808	0.0812	0.0816
**0.2300**	0.1210	0.1215	0.1220	0.1225	0.1230	0.1235	0.1240	0.1245	0.1250	0.1256	0.1261	0.1266
**0.2400**	0.1779	0.1785	0.1792	0.1798	0.1804	0.1811	0.1817	0.1824	0.1830	0.1837	0.1843	0.1850
**0.2500**	0.2476	0.2484	0.2491	0.2499	0.2506	0.2514	0.2522	0.2529	0.2537	0.2545	0.2552	0.2560
**0.2600**	0.3281	0.3289	0.3298	0.3306	0.3314	0.3323	0.3332	0.3340	0.3349	0.3357	0.3366	0.3374
**0.2700**	0.4158	0.4167	0.4176	0.4185	0.4194	0.4203	0.4212	0.4221	0.4230	0.4239	0.4248	0.4257
**0.2800**	0.5064	0.5074	0.5083	0.5092	0.5101	0.5110	0.5119	0.5128	0.5137	0.5146	0.5155	0.5164
**0.2900**	0.5955	0.5964	0.5972	0.5981	0.5990	0.5998	0.6007	0.6016	0.6024	0.6033	0.6041	0.6050
**0.3000**	0.6788	0.6796	0.6804	0.6812	0.6820	0.6827	0.6835	0.6843	0.6851	0.6859	0.6867	0.6875
**0.3100**	0.7531	0.7538	0.7545	0.7552	0.7559	0.7566	0.7572	0.7579	0.7586	0.7593	0.7600	0.7607
**0.3200**	0.8165	0.8171	0.8176	0.8182	0.8188	0.8194	0.8199	0.8205	0.8211	0.8216	0.8222	0.8228
**0.3300**	0.8682	0.8686	0.8691	0.8695	0.8700	0.8705	0.8709	0.8714	0.8718	0.8723	0.8727	0.8732
**0.3400**	0.9085	0.9089	0.9092	0.9096	0.9099	0.9103	0.9106	0.9110	0.9113	0.9117	0.9120	0.9123
**0.3500**	0.9387	0.9390	0.9393	0.9395	0.9398	0.9400	0.9403	0.9405	0.9408	0.9410	0.9413	0.9415
**0.3600**	0.9604	0.9606	0.9608	0.9610	0.9611	0.9613	0.9615	0.9617	0.9618	0.9620	0.9622	0.9624
**0.3700**	0.9754	0.9755	0.9756	0.9757	0.9758	0.9760	0.9761	0.9762	0.9763	0.9764	0.9765	0.9767
**0.3800**	0.9852	0.9853	0.9854	0.9854	0.9855	0.9856	0.9857	0.9857	0.9858	0.9859	0.9860	0.9860
**0.3900**	0.9914	0.9915	0.9915	0.9916	0.9916	0.9917	0.9917	0.9918	0.9918	0.9919	0.9919	0.9920
**0.4000**	0.9952	0.9953	0.9953	0.9953	0.9954	0.9954	0.9954	0.9954	0.9955	0.9955	0.9955	0.9956
**0.4100**	0.9975	0.9975	0.9975	0.9975	0.9975	0.9975	0.9976	0.9976	0.9976	0.9976	0.9976	0.9976
**0.4200**	0.9987	0.9987	0.9987	0.9987	0.9987	0.9987	0.9987	0.9988	0.9988	0.9988	0.9988	0.9988
**0.4300**	0.9994	0.9994	0.9994	0.9994	0.9994	0.9994	0.9994	0.9994	0.9994	0.9994	0.9994	0.9994
**0.4400**	0.9997	0.9997	0.9997	0.9997	0.9997	0.9997	0.9997	0.9997	0.9997	0.9997	0.9997	0.9997
**0.4500**	0.9999	0.9999	0.9999	0.9999	0.9999	0.9999	0.9999	0.9999	0.9999	0.9999	0.9999	0.9999
**0.4600**	0.9999	0.9999	0.9999	0.9999	0.9999	0.9999	0.9999	0.9999	0.9999	0.9999	0.9999	0.9999
**0.4700**	1.0000	1.0000	1.0000	1.0000	1.0000	1.0000	1.0000	1.0000	1.0000	1.0000	1.0000	1.0000

Appendix A: Table of Integrals (continued)

Q distribution of Fisher-Escolà for testing quantum probability transitions. Prepared by *Prof. Dr. Álex Escolà-Gascón*

	Third and fourth decimal places											
$Q_{\text{Fisher-Escolà}}$	**0.0060**	**0.0061**	**0.0062**	**0.0063**	**0.0064**	**0.0065**	**0.0066**	**0.0067**	**0.0068**	**0.0069**	**0.0070**	**0.0071**
**0.1300**	~0	~0	~0	~0	~0	~0	~0	~0	~0	~0	~0	~0
**0.1400**	0.0001	0.0001	0.0001	0.0001	0.0001	0.0001	0.0001	0.0001	0.0001	0.0001	0.0001	0.0001
**0.1500**	0.0003	0.0003	0.0004	0.0004	0.0004	0.0004	0.0004	0.0004	0.0004	0.0004	0.0004	0.0004
**0.1600**	0.0011	0.0011	0.0011	0.0011	0.0011	0.0011	0.0011	0.0011	0.0011	0.0012	0.0012	0.0012
**0.1700**	0.0028	0.0029	0.0029	0.0029	0.0029	0.0030	0.0030	0.0030	0.0030	0.0031	0.0031	0.0031
**0.1800**	0.0067	0.0067	0.0068	0.0069	0.0069	0.0070	0.0070	0.0071	0.0071	0.0072	0.0073	0.0073
**0.1900**	0.0143	0.0144	0.0145	0.0146	0.0147	0.0148	0.0149	0.0150	0.0151	0.0152	0.0153	0.0154
**0.2000**	0.0277	0.0279	0.0280	0.0282	0.0284	0.0286	0.0287	0.0289	0.0291	0.0293	0.0294	0.0296
**0.2100**	0.0494	0.0497	0.0500	0.0503	0.0505	0.0508	0.0511	0.0513	0.0516	0.0519	0.0522	0.0525
**0.2200**	0.0820	0.0823	0.0827	0.0831	0.0835	0.0839	0.0843	0.0847	0.0851	0.0855	0.0859	0.0863
**0.2300**	0.1271	0.1276	0.1281	0.1287	0.1292	0.1297	0.1302	0.1308	0.1313	0.1318	0.1323	0.1329
**0.2400**	0.1856	0.1863	0.1869	0.1876	0.1882	0.1889	0.1895	0.1902	0.1908	0.1915	0.1922	0.1928
**0.2500**	0.2568	0.2575	0.2583	0.2591	0.2598	0.2606	0.2614	0.2622	0.2629	0.2637	0.2645	0.2653
**0.2600**	0.3383	0.3391	0.3400	0.3408	0.3417	0.3426	0.3434	0.3443	0.3451	0.3460	0.3469	0.3477
**0.2700**	0.4266	0.4275	0.4284	0.4293	0.4302	0.4311	0.4320	0.4329	0.4338	0.4347	0.4356	0.4365
**0.2800**	0.5173	0.5182	0.5191	0.5200	0.5209	0.5218	0.5227	0.5236	0.5245	0.5254	0.5263	0.5272
**0.2900**	0.6059	0.6067	0.6076	0.6085	0.6093	0.6102	0.6110	0.6119	0.6127	0.6136	0.6144	0.6153
**0.3000**	0.6882	0.6890	0.6898	0.6906	0.6914	0.6921	0.6929	0.6937	0.6945	0.6952	0.6960	0.6968
**0.3100**	0.7613	0.7620	0.7627	0.7634	0.7640	0.7647	0.7654	0.7660	0.7667	0.7674	0.7680	0.7687
**0.3200**	0.8233	0.8239	0.8244	0.8250	0.8255	0.8261	0.8267	0.8272	0.8278	0.8283	0.8289	0.8294
**0.3300**	0.8736	0.8740	0.8745	0.8749	0.8754	0.8758	0.8763	0.8767	0.8771	0.8776	0.8780	0.8784
**0.3400**	0.9127	0.9130	0.9133	0.9137	0.9140	0.9144	0.9147	0.9150	0.9153	0.9157	0.9160	0.9163
**0.3500**	0.9418	0.9420	0.9423	0.9425	0.9427	0.9430	0.9432	0.9435	0.9437	0.9440	0.9442	0.9444
**0.3600**	0.9625	0.9627	0.9629	0.9631	0.9632	0.9634	0.9636	0.9637	0.9639	0.9641	0.9642	0.9644
**0.3700**	0.9768	0.9769	0.9770	0.9771	0.9772	0.9773	0.9775	0.9776	0.9777	0.9778	0.9779	0.9780
**0.3800**	0.9861	0.9862	0.9863	0.9863	0.9864	0.9865	0.9866	0.9866	0.9867	0.9868	0.9868	0.9869
**0.3900**	0.9920	0.9921	0.9921	0.9922	0.9922	0.9922	0.9923	0.9923	0.9924	0.9924	0.9925	0.9925
**0.4000**	0.9956	0.9956	0.9956	0.9957	0.9957	0.9957	0.9957	0.9958	0.9958	0.9958	0.9958	0.9959
**0.4100**	0.9976	0.9977	0.9977	0.9977	0.9977	0.9977	0.9977	0.9978	0.9978	0.9978	0.9978	0.9978
**0.4200**	0.9988	0.9988	0.9988	0.9988	0.9988	0.9988	0.9988	0.9989	0.9989	0.9989	0.9989	0.9989
**0.4300**	0.9994	0.9994	0.9994	0.9994	0.9994	0.9994	0.9994	0.9994	0.9994	0.9994	0.9995	0.9995
**0.4400**	0.9997	0.9997	0.9997	0.9997	0.9997	0.9997	0.9997	0.9997	0.9997	0.9997	0.9997	0.9997
**0.4500**	0.9999	0.9999	0.9999	0.9999	0.9999	0.9999	0.9999	0.9999	0.9999	0.9999	0.9999	0.9999
**0.4600**	0.9999	0.9999	0.9999	0.9999	0.9999	0.9999	0.9999	0.9999	1.0000	1.0000	1.0000	1.0000
**0.4700**	1.0000	1.0000	1.0000	1.0000	1.0000	1.0000	1.0000	1.0000	1.0000	1.0000	1.0000	1.0000

Appendix A: Table of Integrals (continued)

Q distribution of Fisher-Escolà for testing quantum probability transitions. Prepared by *Prof. Dr. Álex Escolà-Gascón*

	Third and fourth decimal places											
$Q_{\text{Fisher-Escolà}}$	**0.0072**	**0.0073**	**0.0074**	**0.0075**	**0.0076**	**0.0077**	**0.0078**	**0.0079**	**0.0080**	**0.0081**	**0.0082**	**0.0083**
**0.1300**	~0	~0	~0	~0	~0	~0	~0	~0	~0	~0	~0	~0
**0.1400**	0.0001	0.0001	0.0001	0.0001	0.0001	0.0001	0.0001	0.0001	0.0001	0.0001	0.0001	0.0001
**0.1500**	0.0004	0.0004	0.0004	0.0004	0.0004	0.0004	0.0004	0.0004	0.0004	0.0004	0.0004	0.0005
**0.1600**	0.0012	0.0012	0.0012	0.0012	0.0012	0.0013	0.0013	0.0013	0.0013	0.0013	0.0013	0.0013
**0.1700**	0.0032	0.0032	0.0032	0.0032	0.0033	0.0033	0.0033	0.0034	0.0034	0.0034	0.0034	0.0035
**0.1800**	0.0074	0.0074	0.0075	0.0075	0.0076	0.0077	0.0077	0.0078	0.0079	0.0079	0.0080	0.0080
**0.1900**	0.0155	0.0156	0.0157	0.0158	0.0160	0.0161	0.0162	0.0163	0.0164	0.0165	0.0166	0.0168
**0.2000**	0.0298	0.0300	0.0302	0.0304	0.0305	0.0307	0.0309	0.0311	0.0313	0.0315	0.0317	0.0319
**0.2100**	0.0527	0.0530	0.0533	0.0536	0.0539	0.0542	0.0544	0.0547	0.0550	0.0553	0.0556	0.0559
**0.2200**	0.0867	0.0871	0.0875	0.0879	0.0883	0.0887	0.0891	0.0895	0.0899	0.0904	0.0908	0.0912
**0.2300**	0.1334	0.1339	0.1345	0.1350	0.1356	0.1361	0.1366	0.1372	0.1377	0.1383	0.1388	0.1394
**0.2400**	0.1935	0.1942	0.1948	0.1955	0.1962	0.1968	0.1975	0.1982	0.1989	0.1995	0.2002	0.2009
**0.2500**	0.2660	0.2668	0.2676	0.2684	0.2692	0.2700	0.2707	0.2715	0.2723	0.2731	0.2739	0.2747
**0.2600**	0.3486	0.3495	0.3503	0.3512	0.3521	0.3529	0.3538	0.3547	0.3555	0.3564	0.3573	0.3581
**0.2700**	0.4374	0.4383	0.4393	0.4402	0.4411	0.4420	0.4429	0.4438	0.4447	0.4456	0.4465	0.4474
**0.2800**	0.5281	0.5290	0.5299	0.5308	0.5317	0.5326	0.5335	0.5344	0.5353	0.5362	0.5371	0.5380
**0.2900**	0.6162	0.6170	0.6179	0.6187	0.6196	0.6204	0.6213	0.6221	0.6230	0.6238	0.6246	0.6255
**0.3000**	0.6975	0.6983	0.6991	0.6999	0.7006	0.7014	0.7022	0.7029	0.7037	0.7044	0.7052	0.7060
**0.3100**	0.7694	0.7700	0.7707	0.7714	0.7720	0.7727	0.7733	0.7740	0.7747	0.7753	0.7760	0.7766
**0.3200**	0.8300	0.8305	0.8311	0.8316	0.8321	0.8327	0.8332	0.8338	0.8343	0.8348	0.8354	0.8359
**0.3300**	0.8789	0.8793	0.8797	0.8802	0.8806	0.8810	0.8814	0.8819	0.8823	0.8827	0.8831	0.8835
**0.3400**	0.9167	0.9170	0.9173	0.9176	0.9180	0.9183	0.9186	0.9189	0.9192	0.9196	0.9199	0.9202
**0.3500**	0.9447	0.9449	0.9451	0.9454	0.9456	0.9458	0.9461	0.9463	0.9465	0.9468	0.9470	0.9472
**0.3600**	0.9646	0.9647	0.9649	0.9650	0.9652	0.9654	0.9655	0.9657	0.9659	0.9660	0.9662	0.9663
**0.3700**	0.9781	0.9782	0.9783	0.9784	0.9786	0.9787	0.9788	0.9789	0.9790	0.9791	0.9792	0.9793
**0.3800**	0.9870	0.9871	0.9871	0.9872	0.9873	0.9873	0.9874	0.9875	0.9875	0.9876	0.9877	0.9877
**0.3900**	0.9925	0.9926	0.9926	0.9927	0.9927	0.9928	0.9928	0.9928	0.9929	0.9929	0.9930	0.9930
**0.4000**	0.9959	0.9959	0.9959	0.9960	0.9960	0.9960	0.9960	0.9961	0.9961	0.9961	0.9961	0.9962
**0.4100**	0.9978	0.9978	0.9979	0.9979	0.9979	0.9979	0.9979	0.9979	0.9979	0.9980	0.9980	0.9980
**0.4200**	0.9989	0.9989	0.9989	0.9989	0.9989	0.9989	0.9989	0.9989	0.9990	0.9990	0.9990	0.9990
**0.4300**	0.9995	0.9995	0.9995	0.9995	0.9995	0.9995	0.9995	0.9995	0.9995	0.9995	0.9995	0.9995
**0.4400**	0.9997	0.9998	0.9998	0.9998	0.9998	0.9998	0.9998	0.9998	0.9998	0.9998	0.9998	0.9998
**0.4500**	0.9999	0.9999	0.9999	0.9999	0.9999	0.9999	0.9999	0.9999	0.9999	0.9999	0.9999	0.9999
**0.4600**	1.0000	1.0000	1.0000	1.0000	1.0000	1.0000	1.0000	1.0000	1.0000	1.0000	1.0000	1.0000
**0.4700**	1.0000	1.0000	1.0000	1.0000	1.0000	1.0000	1.0000	1.0000	1.0000	1.0000	1.0000	1.0000

Appendix A: Table of Integrals (continued)

Q distribution of Fisher-Escolà for testing quantum probability transitions. Prepared by *Prof. Dr. Álex Escolà-Gascón*

Third and fourth decimal places

$Q_{\text{Fisher-Escolà}}$	0.0084	0.0085	0.0086	0.0087	0.0088	0.0089	0.0090	0.0091	0.0092	0.0093	0.0094	0.0095
**0.1300**	~0	~0	~0	~0	~0	~0	~0	~0	~0	~0	~0	~0
**0.1400**	0.0001	0.0001	0.0001	0.0001	0.0001	0.0001	0.0001	0.0001	0.0001	0.0001	0.0001	0.0002
**0.1500**	0.0005	0.0005	0.0005	0.0005	0.0005	0.0005	0.0005	0.0005	0.0005	0.0005	0.0005	0.0005
**0.1600**	0.0014	0.0014	0.0014	0.0014	0.0014	0.0014	0.0014	0.0015	0.0015	0.0015	0.0015	0.0015
**0.1700**	0.0035	0.0035	0.0036	0.0036	0.0036	0.0037	0.0037	0.0037	0.0038	0.0038	0.0038	0.0039
**0.1800**	0.0081	0.0082	0.0082	0.0083	0.0084	0.0084	0.0085	0.0086	0.0086	0.0087	0.0088	0.0088
**0.1900**	0.0169	0.0170	0.0171	0.0172	0.0173	0.0175	0.0176	0.0177	0.0178	0.0179	0.0181	0.0182
**0.2000**	0.0321	0.0322	0.0324	0.0326	0.0328	0.0330	0.0332	0.0334	0.0336	0.0338	0.0340	0.0342
**0.2100**	0.0562	0.0565	0.0568	0.0571	0.0574	0.0577	0.0580	0.0583	0.0586	0.0589	0.0592	0.0595
**0.2200**	0.0916	0.0920	0.0924	0.0929	0.0933	0.0937	0.0941	0.0945	0.0950	0.0954	0.0958	0.0963
**0.2300**	0.1399	0.1405	0.1410	0.1416	0.1421	0.1427	0.1432	0.1438	0.1444	0.1449	0.1455	0.1461
**0.2400**	0.2016	0.2023	0.2029	0.2036	0.2043	0.2050	0.2057	0.2064	0.2071	0.2078	0.2085	0.2091
**0.2500**	0.2755	0.2763	0.2771	0.2779	0.2787	0.2795	0.2803	0.2811	0.2819	0.2827	0.2835	0.2843
**0.2600**	0.3590	0.3599	0.3608	0.3616	0.3625	0.3634	0.3642	0.3651	0.3660	0.3669	0.3678	0.3686
**0.2700**	0.4483	0.4492	0.4501	0.4510	0.4519	0.4529	0.4538	0.4547	0.4556	0.4565	0.4574	0.4583
**0.2800**	0.5389	0.5398	0.5407	0.5416	0.5425	0.5434	0.5443	0.5452	0.5461	0.5470	0.5479	0.5488
**0.2900**	0.6263	0.6272	0.6280	0.6289	0.6297	0.6306	0.6314	0.6322	0.6331	0.6339	0.6348	0.6356
**0.3000**	0.7067	0.7075	0.7082	0.7090	0.7097	0.7105	0.7112	0.7120	0.7128	0.7135	0.7142	0.7150
**0.3100**	0.7773	0.7779	0.7786	0.7792	0.7799	0.7805	0.7811	0.7818	0.7824	0.7831	0.7837	0.7843
**0.3200**	0.8364	0.8370	0.8375	0.8380	0.8386	0.8391	0.8396	0.8401	0.8407	0.8412	0.8417	0.8422
**0.3300**	0.8840	0.8844	0.8848	0.8852	0.8856	0.8860	0.8865	0.8869	0.8873	0.8877	0.8881	0.8885
**0.3400**	0.9205	0.9208	0.9211	0.9214	0.9218	0.9221	0.9224	0.9227	0.9230	0.9233	0.9236	0.9239
**0.3500**	0.9474	0.9477	0.9479	0.9481	0.9483	0.9486	0.9488	0.9490	0.9492	0.9495	0.9497	0.9499
**0.3600**	0.9665	0.9666	0.9668	0.9670	0.9671	0.9673	0.9674	0.9676	0.9677	0.9679	0.9680	0.9682
**0.3700**	0.9794	0.9795	0.9796	0.9797	0.9798	0.9799	0.9800	0.9801	0.9802	0.9803	0.9804	0.9805
**0.3800**	0.9878	0.9879	0.9879	0.9880	0.9881	0.9881	0.9882	0.9883	0.9883	0.9884	0.9884	0.9885
**0.3900**	0.9930	0.9931	0.9931	0.9932	0.9932	0.9932	0.9933	0.9933	0.9934	0.9934	0.9934	0.9935
**0.4000**	0.9962	0.9962	0.9962	0.9963	0.9963	0.9963	0.9963	0.9964	0.9964	0.9964	0.9964	0.9964
**0.4100**	0.9980	0.9980	0.9980	0.9980	0.9980	0.9981	0.9981	0.9981	0.9981	0.9981	0.9981	0.9981
**0.4200**	0.9990	0.9990	0.9990	0.9990	0.9990	0.9990	0.9990	0.9990	0.9990	0.9990	0.9991	0.9991
**0.4300**	0.9995	0.9995	0.9995	0.9995	0.9995	0.9995	0.9995	0.9995	0.9995	0.9995	0.9995	0.9995
**0.4400**	0.9998	0.9998	0.9998	0.9998	0.9998	0.9998	0.9998	0.9998	0.9998	0.9998	0.9998	0.9998
**0.4500**	0.9999	0.9999	0.9999	0.9999	0.9999	0.9999	0.9999	0.9999	0.9999	0.9999	0.9999	0.9999
**0.4600**	1.0000	1.0000	1.0000	1.0000	1.0000	1.0000	1.0000	1.0000	1.0000	1.0000	1.0000	1.0000
**0.4700**	1.0000	1.0000	1.0000	1.0000	1.0000	1.0000	1.0000	1.0000	1.0000	1.0000	1.0000	1.0000

Appendix A: Table of Integrals (continued)

Q distribution of Fisher-Escolà for testing quantum probability transitions. Prepared by *Prof. Dr. Álex Escolà-Gascón*

Third and fourth decimal places

$Q_{\text{Fisher-Escolà}}$	**0.0084**	**0.0085**	**0.0086**	**0.0087**
**0.1300**	~0	~0	~0	~0
**0.1400**	0.0001	0.0001	0.0001	0.0001
**0.1500**	0.0005	0.0005	0.0005	0.0005
**0.1600**	0.0014	0.0014	0.0014	0.0014
**0.1700**	0.0035	0.0035	0.0036	0.0036
**0.1800**	0.0081	0.0082	0.0082	0.0083
**0.1900**	0.0169	0.0170	0.0171	0.0172
**0.2000**	0.0321	0.0322	0.0324	0.0326
**0.2100**	0.0562	0.0565	0.0568	0.0571
**0.2200**	0.0916	0.0920	0.0924	0.0929
**0.2300**	0.1399	0.1405	0.1410	0.1416
**0.2400**	0.2016	0.2023	0.2029	0.2036
**0.2500**	0.2755	0.2763	0.2771	0.2779
**0.2600**	0.3590	0.3599	0.3608	0.3616
**0.2700**	0.4483	0.4492	0.4501	0.4510
**0.2800**	0.5389	0.5398	0.5407	0.5416
**0.2900**	0.6263	0.6272	0.6280	0.6289
**0.3000**	0.7067	0.7075	0.7082	0.7090
**0.3100**	0.7773	0.7779	0.7786	0.7792
**0.3200**	0.8364	0.8370	0.8375	0.8380
**0.3300**	0.8840	0.8844	0.8848	0.8852
**0.3400**	0.9205	0.9208	0.9211	0.9214
**0.3500**	0.9474	0.9477	0.9479	0.9481
**0.3600**	0.9665	0.9666	0.9668	0.9670
**0.3700**	0.9794	0.9795	0.9796	0.9797
**0.3800**	0.9878	0.9879	0.9879	0.9880
**0.3900**	0.9930	0.9931	0.9931	0.9932
**0.4000**	0.9962	0.9962	0.9962	0.9963
**0.4100**	0.9980	0.9980	0.9980	0.9980
**0.4200**	0.9990	0.9990	0.9990	0.9990
**0.4300**	0.9995	0.9995	0.9995	0.9995
**0.4400**	0.9998	0.9998	0.9998	0.9998
**0.4500**	0.9999	0.9999	0.9999	0.9999
**0.4600**	1.0000	1.0000	1.0000	1.0000
**0.4700**	1.0000	1.0000	1.0000	1.0000
